# Single-blinded, randomised, parallel-group, controlled trial comparing the efficacy and cost-effectiveness of therapist- and self-guided internet-delivered behavioural activation versus treatment as usual for adolescents with mild to moderate depression: study protocol

**DOI:** 10.1136/bmjopen-2023-083507

**Published:** 2024-10-15

**Authors:** Rebecca Andersson, Sarah Vigerland, Fabian Lenhard, Johan Ahlen, Matteo Bottai, David Mataix-Cols, Eva Serlachius

**Affiliations:** 1 Centre for Psychiatry Research, Department of Clinical Neuroscience, Karolinska Institutet, & Stockholm Health Care Services, Region Stockholm, CAP Research Centre, Stockholm, Sweden; 2 The Centre for Epidemiology and Community Medicine, Region Stockholm, Sweden, Sweden; 3 Department of Global Public Health, Karolinska Institutet, Stockholm, Sweden; 4 Division of Biostatistics, Institute of Environmental Medicine, Karolinska Institutet, Stockholm, Sweden; 5 Department of Clinical Sciences, Faculty of Medicine, Lund University, Lund, Sweden; 6 Department of Clinical Neuroscience, Karolinska Institutet, Stockholm, Sweden

**Keywords:** Depression & mood disorders, Adolescents, Clinical Trial, Health economics, Child & adolescent psychiatry

## Abstract

**Introduction:**

The number of adolescents seeking professional help for depression is increasing and, despite advances in treatment, large unmet treatment needs remain. In the current protocol, we describe the design and methodology of a randomised controlled trial (RCT) to evaluate the clinical efficacy of two forms of internet-delivered behavioural activation (I-BA), with and without therapist support, in reducing depressive symptoms, compared with treatment as usual (TAU). Secondary objectives include examining the 12-month maintenance of the treatment effects and conducting a health economic evaluation of the interventions.

**Methods and analysis:**

In this single-blinded RCT, we aim to include 215 participants aged 13–17 years with mild to moderate depression who will be randomised (1:1:1 ratio) to 10 weeks of either therapist-guided or self-guided I-BA, or TAU provided by regular mental health clinics. Data will be collected at baseline, weekly for the initial 10 weeks, post-treatment and at 3 and 12-month follow-ups. The primary endpoint is the 3-month follow-up. The primary outcome is blinded clinician-rated severity of depressive symptoms, measured by the Children’s Depression Rating Scale-Revised. Treatment response is defined as a score of ‘Much improved’ or ‘Very much improved’ on the Clinical Global Impression-Improvement Scale, administered at the primary endpoint. Outcome assessors will be blinded to treatment conditions at all assessment points. A health economic evaluation of I-BA will be performed, both in the short term (primary endpoint) and the long term (12-month follow-up).

**Ethics and dissemination:**

Ethical approval was obtained from the Swedish Ethical Review Authority in June 2021. The final participant was enrolled on 3 May 2024 and expected to reach the primary endpoint by November 2024. The results of this study will be disseminated through publication in peer-reviewed journals, presented at conferences and communicated to healthcare providers and the public.

**Trial registration number:**

NCT04977856.

Rebecca Andersson^1^, Sarah Vigerland^1^, Fabian Lenhard ^1^, Johan Ahlen^2, 3^, Matteo Bottai^4^, David Mataix-Cols^1,6^, Eva Serlachius^5, 6^

**STRENGTHS AND LIMITATIONS OF THIS STUDY**

- Simultaneous evaluation of two versions of internet-delivered behavioural activation: therapist guided and self guided, which enables isolation of the contribution of therapist support.
- Use of an active and ecologically valid comparator (eg, treatment as usual).
- Assessments include multiple informant reports and blinded follow-ups that continue for up to 12 months.
- Both therapists and participating families are aware of their assigned treatments.

## Introduction

 Depression is a leading cause of disability worldwide and a global health priority.^1^ Adolescence is a vulnerable period for developing depression, and the prevalence has risen sharply over the past decade.^2^ For instance, in the USA, the 1-year prevalence of broadly defined depression in adolescents increased from 8.3% to 12.9% between 2011 and 2016.^3^ A similar increasing trend is seen in other countries including the UK^4^ and Sweden.^5^ Depression in adolescence is associated with several adverse outcomes, such as a risk for recurrence of depression,^6^ development of comorbid psychiatric and somatic conditions,^7 8^ substantially increased risk of suicide,^9^ as well as increased psychosocial impairments in adulthood.^10^ Although treatment for adolescent depression can reverse many of these negative effects, only a minority access effective treatments.^11^ Thus, there is an urgent need to improve treatment access for adolescents with depression.

One viable solution is to deliver psychological treatments digitally, a format that resonates with the lifestyles and preferences of young people. Digital therapies potentially have important advantages over traditional face-to-face treatments, including cost-effectiveness, increased accessibility, limited therapist drift and reduced stigma associated with traditional mental health services. In a recent review of internet-delivered interventions for adolescents,^12^ 10 studies on depression were identified. Among them, only two were rated as having ‘good study quality’, namely Ip *et al*^13^ and Stasiak *et al*.^14^ However, Ip *et al*’s study was conducted with a subclinical sample only, and Stasiak *et al* had a relatively small study sample. To sum, this review highlights the need for high-quality randomised controlled trials (RCTs) that also incorporate health economic evaluations.

Internet-delivered interventions can be provided with or without therapist support. Most meta-analyses show that guided interventions are generally associated with greater improvement,^15^,^17^ although Pennant *et al*^18^ did not find that therapist input had any significant impact on effectiveness of treating depression in young people. Among adults with milder problems, self-guided cognitive–behavioural therapy (CBT) was as effective as guided CBT.^15^ If internet-delivered CBT (ICBT) could be unguided, without sacrificing efficacy and safety, it would be much easier to disseminate.

As a preparatory step for this study, we conducted a randomised feasibility trial (n=32) of therapist-guided and self-guided internet-delivered behavioural activation (I-BA), compared with treatment as usual (TAU).^19^ The study demonstrated positive results in terms of participant retention, treatment adherence, overall safety, as well as preliminary clinical efficacy. Both therapist-guided and self-guided I-BA (Cohen’s d=2.43 and 2.23, respectively), but not TAU (Cohen’s d=0.95), showed statistically significant changes on assessor-rated depressive symptoms, with large within-group effect sizes. In summary, this study suggests the feasibility of conducting a large-scale trial to evaluate the efficacy and cost-effectiveness of therapist-guided and self-guided I-BA.

Behavioural activation (BA) is an evidence-based treatment for adults with depression^20 21^ and empirical support is growing for adolescents.^22^,^24^ However, BA has yet to be evaluated in an adequately powered RCT. Unlike classic CBT for depression, BA does not include cognitive restructuring,^25^ yet it seems to be equally effective.^26 27^ Results from our feasibility study suggested that both therapist-guided and self-guided I-BA are acceptable and potentially efficacious treatments for adolescents with depression, and that TAU is a reasonable and ethically acceptable control condition.

In this paper, we describe the study protocol of a single-blinded, fully powered RCT with the primary objective to compare the relative clinical efficacy of therapist-guided and self-guided I-BA versus TAU for reducing assessor-rated depressive symptoms in adolescents with mild to moderate major depression. The secondary objectives are to establish the maintenance of treatment effects at 12 months post-treatment and to conduct a health economic evaluation of therapist-guided and self-guided I-BA, both short term (primary endpoint) and long term (12-month follow-up). Based on the feasibility study, the main hypotheses are that both forms of I-BA will be superior to TAU in terms of efficacy and cost-effectiveness.

## Methods and analysis

### Study design and setting

**Figure 1:**
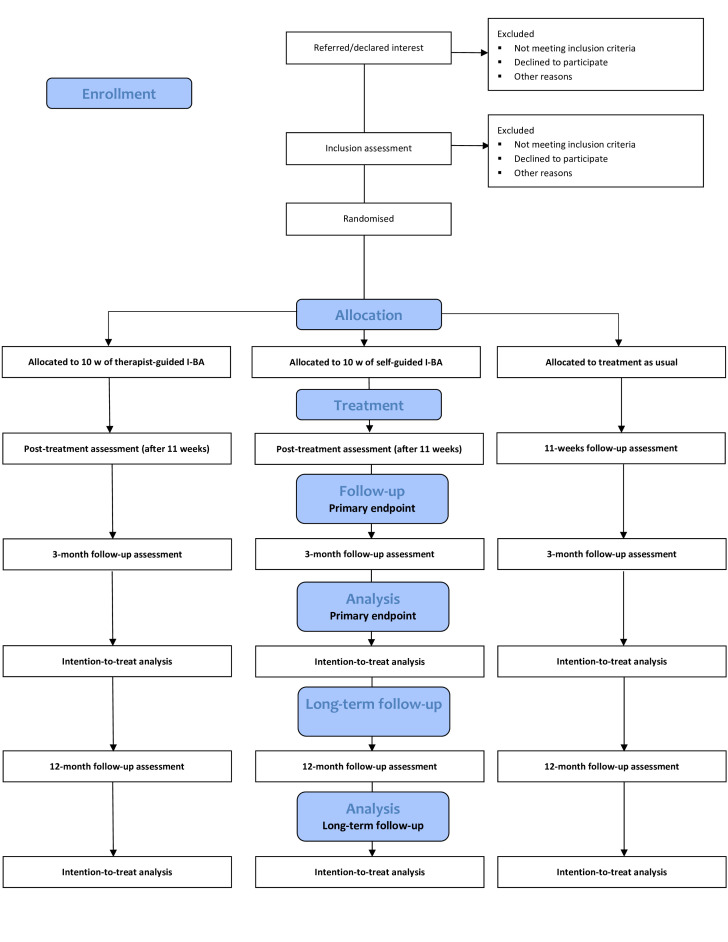
Consolidated Standards of Reporting Trials (CONSORT) 2010 flow chart. I-BA, internet-delivered behavioural activation.

This study is a single-blinded, parallel-group, three-arm, randomised controlled superiority study with a 1:1:1 allocation ratio of therapist-guided I-BA, self-guided I-BA and TAU for adolescents with mild to moderate major depression. The full study protocol is available in online supplemental file 1. No major changes in the design have been made after the registration and subsequent start of the trial. The study design is depicted in figure 1. This study protocol follows the Standard Protocol Items: Recommendations for Interventional Trials reporting guideline for clinical trials.^28^

The study will be conducted at two specialist outpatient Child and Adolescent Mental Health Services (CAMHS) sites in Stockholm (the investigational site) and Lund, Sweden. Participants will be recruited nationally from both urban and rural areas across Sweden.

### Participants

#### Eligibility criteria

Inclusion criteria:

- Aged 13–17 years (inclusive).
- A diagnosis of mild to moderate major depression based on the 5th Edition of the Diagnostic and Statistical Manual of Mental Disorders (DSM-5).^29^
- Willing to be randomised to either of the three treatment arms.
- Both adolescent and parent fluent in Swedish.
- Regular access to the internet via a smartphone or a computer.
- If using medication with antidepressants, central stimulants or neuroleptics, it has to be unchanged at least 6 weeks prior to inclusion.
- At least one available caregiver/parent (hereafter referred to as parent) to support the adolescent throughout the treatment.

Exclusion criteria:

- The presence of psychiatric problems requiring immediate treatment (eg, high risk of suicide, psychosis, severe self-injury, bipolar disorder, clinical eating disorder, alcohol/substance abuse).
- Social problems requiring immediate action (eg, ongoing abuse in the family, high and prolonged absence from school).
- Previous psychological treatment for major depressive disorder (MDD; CBT, interpersonal psychotherapy or BA) for a minimum of at least three sessions within the last 12 months prior to assessment.
- Current use of benzodiazepines.
- Ongoing psychological treatment for any psychiatric disorder.

#### Recruitment and procedures

Participants from all over Sweden will be able to either self-refer via an online registration form at the study website or be referred by a clinician to our specialist CAMHS clinics in Stockholm or Lund. The study will be advertised to healthcare services, patient organisations, the local press and social media.

Following self-referral or clinician referral, the participants will be assigned a screening ID and be contacted by phone by a member of the research team to provide study information and conduct a preliminary eligibility screening. In the Lund recruitment site, recent information from medical records will be used to assist in the screening of the eligibility criteria.

If the applicant is interested and potentially eligible, the adolescent and at least one of the parents will be invited for an inclusion assessment for more thorough study eligibility, face to face at one of the two sites or via video for families who cannot travel to the clinic (no travel expenses will be reimbursed). Prior to the inclusion assessment, the family will be sent a written informed consent form, to be signed on paper or digitally through a secure platform. Data collection will not start before informed consent has been signed by the adolescent and the parent/s.

During the assessment visit, a clinical psychologist will (a) verify the diagnosis of MDD according to DSM-5 criteria; (b) assess the current severity level of depression using the Children’s Depression Rating Scale-Revised (CDRS-R); (c) assess psychiatric comorbidity; and (d) collect information on medical history and sociodemographic variables. If eligible, the adolescent will be offered participation in the study. Excluded participants who still require medical care will be referred to other appropriate services.

Included participants will then fill in the baseline adolescent-reported and parent-reported baseline questionnaires online (see the ‘Outcome measures’ section), and then be randomised and assigned a study ID (week 0). Within a week, patients allocated to the I-BA arms will start treatment, and participants allocated to TAU receive a referral to their local CAMHS or primary care clinic.

Assessments will occur at predefined time points after allocation and are independent of the treatment progress. Blinded assessments will be conducted at post-treatment (week 11), 3-month follow-up (primary endpoint) and 1-year follow-up. Setting the primary endpoint at the 3-month follow-up increases the likelihood that participants assigned to TAU will have received treatment. Additionally, previous ICBT trials, including our pilot study,^19^ have shown continued improvement from post-treatment to 3-month follow-up. Long-term effects will be investigated with a 1-year follow-up. Adolescent-reported and parent-reported measures will be completed online at all assessment points. Adolescent-reported questionnaires on depressive symptom severity will also be administered weekly during the first 10 weeks of treatment for the I-BA groups (and 10 weeks after the referral is sent for TAU). Changes in psychotropic medication and additional psychological treatment will be monitored and documented throughout the study period.

#### Safety procedures

A thorough diagnostic and mental health status screening before enrolling participants will prevent patients with more immediate psychiatric needs or risks from being included in the study and instead referred to appropriate care. All participants will have a basic crisis plan, including contact details to their parent (or another adult), and information about emergency services, should they experience suicidal thoughts. Participants will also complete a brief measure of depressive symptoms (Quick Inventory of Depressive Symptomatology, Adolescent version (QIDS-A17), see the ‘Measures’ section) weekly during the intervention, allowing suicidal ideation to be monitored and, if necessary, assessed by a research team member by telephone. If further psychiatric assessments or preventive measures are needed, the study team will refer the patient to a psychiatric emergency department.

Further, weekly multidisciplinary team meetings will be held discussing participants’ progress focusing on safety issues. Adverse events will be carefully monitored and reported until the primary endpoint. Where necessary, the participant’s local primary or specialist care unit will be informed about the adverse event.

### Randomisation and allocation concealment

The allocation sequence was generated using block randomisation, with 15 random sets of blocks of six and nine, respectively, by an independent party, the Karolinska Trial Alliance (KTA).^30^ To minimise expectations, participants will only receive concise information about the three treatment arms, while specific details about the content and treatment rationale for BA will not be revealed. Randomisation will be conducted from one site only (the Stockholm hub). Several assigned research team members will be responsible for enrolling and randomising participants through an online automated system for clinical trials (Alea), monitored by the KTA.

Treatment allocation will be blinded for outcome assessors, the statisticians, the health economists and the principal investigator (ES), but not for the project manager (RA), the participants or therapists. Blinded assessors consist of three licensed psychologists and three psychology students (completed the first 3 years of the psychology programme). The students have continuous access to senior colleagues for guidance and support.

To ensure blinding integrity, participants are explicitly asked at follow-up assessments not to disclose the treatment they have received. In the event of accidental unblinding, a new assessor will rerate the participant’s main outcome measures score based on the recording from the interview. Blinding integrity will be checked at each assessment point by asking blinded assessors to guess each participant’s group allocation and indicate the reasons for their guesses (eg, totally random guess, impression of improvement, etc).^31^

### Interventions

#### Therapist-guided and self-guided I-BA

The specific I-BA protocol was developed and adapted to an online format for this study and was inspired by previous BA protocols.^32 33^ BA typically includes treatment rationale and psychoeducation, activity monitoring and scheduling, values and goal assessments, and skills training in problem-solving and communication skills, relaxation techniques and relapse prevention. BA also targets rumination and avoidance.^34^

Although different BA protocols include and emphasise different components, activity monitoring and scheduling are always present.^35^ In our protocol, we included all the previously mentioned BA components except relaxation. Sleep hygiene was added to the BA protocol because sleep problems are common in depression^36^ that are often addressed in face-to-face BA.^37^

The two I-BA treatments will be delivered via a secure online platform, each consisting of eight modules of age-appropriate texts, animations, videos and various exercises delivered over 10 weeks. Each module takes approximately 30–60 min to complete. The first four modules introduce the key components of BA (ie, scheduling of values-based activities and targeting avoidance behaviours). Between each module, both adolescents and parents will be assigned homework, such as using an activity journal to help plan and evaluate scheduled activities. Table 1 provides an overview of the contents and online supplemental file 2 shows screenshots of the I-BA interventions.

**Table 1:** An overview of the treatment content of I-BA

Module	Adolescent intervention	Parent course
1	Psychoeducation and activity monitoring	Psychoeducation about depression and common parental traps
2	Values assessment, treatment goals and values-based activation	Validating your adolescent’s experiences
3	Continued activation. Psychoeducation about sleep	Spending positive time with your adolescent
4	Continued activation. Overcoming avoidance	Avoiding and managing conflicts
5	Continued activation. Shifting focus to the present situation	Taking care of yourself as a parent to a depressed adolescent
6	Continued activation. Problem-solving	Collaborative problem-solving
7	Repetition	Repetition
8	Maintenance, relapse prevention and evaluation	Maintenance, relapse prevention and evaluation

I-BAinternet-delivered behavioural activation

In the therapist-guided I-BA arm, the participants have weekly asynchronous contact with a therapist via written messages within the platform. Therapists in the trial must be licensed psychologists with CBT training and specific training to deliver the I-BA intervention. All therapists participate in weekly peer supervision sessions.

The therapists log in at least every other day during workdays to provide feedback, answer questions and, if needed, prompt the participants to complete the next module. The therapists will be recommended to spend around 20–30 min per family per week. Occasional phone calls will be added when deemed necessary, for example, to help with engagement in the treatment. The content of the self-guided I-BA programme is identical to the therapist-guided version, except that the participants do not have access to any therapist support.

Both conditions of I-BA in this study include a parallel eight-module course for parents (see table 1 for details), accessed through separate login accounts. Involving parents is a common BA adaptation for young people, and parents are educated on how to encourage the young person to complete scheduled activities.^35^ This parent course is based on CBT strategies commonly used in parent training programmes^38^ such as praise and other forms of positive parenting skills aiming at strengthening the relationship between the parent and the adolescent.

#### Comparator (TAU)

Participants randomised to TAU will be referred to regular mental health services within either primary or secondary care (CAMHS). The clinic providing TAU determines what kind of treatment the adolescent will be offered and thus these participants are free to receive any treatment, for instance, psychological, pharmacological or a combination of both.

### Measures

An overview of all measures, assessment points and informants is shown in table 2.

**Table 2:** SPIRIT 2013 schedule of enrolment, interventions and assessments

Measures	Study period							
	Enrolment	Allocation	Post-allocation					
	Baseline		0 week	3 weeks	5 weeks	Post	3FU	12FU
**Enrolment**								
Eligibility screen	X							
Informed consent	X							
Allocation		X						
**Interventions**								
Therapist-guided I-BA			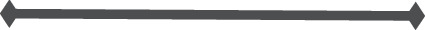					
Self-guided I-BA			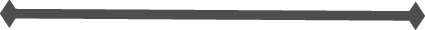					
Comparator*			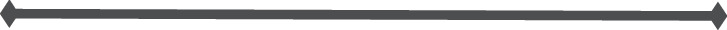					
**Assessments**								
*Baseline measures*								
Demographic data (clinician and parent)	X							
MINI-KID	X							
DSHI-Y-7								
*Assessor-rated*								
Blindness checks						X	X	X
CDRS-R	X					X	X	X
CGAS	X					X	X	X
CGI-I						X	X	X
CGI-S	X					X	X	X
Therapist time					X			
iiPAS						X		
*Self-ratedand parentrated*								
QIDS-17†	X					X	X	X
ARI (self only)	X					X	X	X
ASA (self only)	X					X	X	X
WSAS	X					X	X	X
KIDSCREEN-10	X					X	X	X
RCADS-S	X					X	X	X
ISI (self only)	X					X	X	X
CSQ						X		
Treatment credibility				X				
NEQ-20						X	X	
Need for further treatment							X	
Co-occurring interventions (self only)						X	X	X
BADS-S† (self only)	X					X	X	X
EEAC (parent only)	X					X	X	X
TiC-P (parent only)	X					X	X	X

Comparator is treatment as usual (TAU).

KIDSCREEN-10 is a measure for general health-related quality of life.

0 week refers to the 0 week into treatment, the equivalent of the treatment start/referral sent to TAU.

3 weeks–5 weeks refers to assessment points 3–5 weeks into treatment.

3FU–12FU refers to assessment points 3–12 months after the end of treatment.

* The time period for interventions provided to participants in TAU is unknown to the research team, but theoretically they could start as early as week 0 and be provided until 3FU or beyond.

† Also assessed weekly during the first 10 weeks after allocation.

ARIAffective Reactivity IndexASAAnhedonia Scale for AdolescentsBADS-SBehavioral Activation of Depression Scale-Short formCDRS-RChildren’s Depression Rating Scale-RevisedCGASChildren’s Global Assessment ScaleCGI-IClinical Global Impression-Improvement ScaleCGI-SClinical Global Impression-Severity ScaleCSQClient Satisfaction QuestionnaireDSHI-Y-77-item Deliberate Self-Harm Inventory for YouthEEACExpressed Emotion Adjective Checklist3FU3-month follow-up12FU12-month follow-upI-BAinternet-delivered behavioural activation for adolescents with depressioniiPASInternet Intervention Patient Adherence ScaleISIInsomnia Severity IndexMINI-KIDMini-International Neuropsychiatric Interview for Children and AdolescentsNEQ-2020-item Negative Effects QuestionnaireQIDS-17Quick Inventory of Depressive SymptomatologyRCADS-SRevised Children’s Anxiety and Depression Scale-Short version, anxiety subscalesSPIRITStandard Protocol Items: Recommendations for Interventional TrialsTiC-PTrimbos/iMTA questionnaire for Costs associated with Psychiatric illnessWSASWork and Social Adjustment Scale

#### Baseline measures

The *Mini-International Neuropsychiatric Interview for Children and Adolescents* (MINI-KID)^39^ is administered during the initial assessment to screen for the primary diagnosis of MDD and for psychiatric comorbidities. Assessment of suicide risk is based on all available information, including the suicidality sections of the MINI-KID and CDRS-R collected at the inclusion assessment visit. To assess non-suicidal self-injury, the *7-item Deliberate Self-Harm Inventory for Youth*^40^ is used. Adolescent demographic and clinical data (eg, age, gender, current and previous medications and previous psychological treatment) are collected at the initial assessment, and data from the parents are collected through an online questionnaire.

#### Primary outcome measure

The primary outcome measure is the total score on the *CDRS-R*,^41^ a semistructured clinical interview used to assess depressive symptom severity in youth depression. The total score is the sum of 17 items; total range of 17–113, with higher ratings reflecting greater severity. To minimise measurement errors, all assessors will be extensively trained by the project manager (RA; see online supplemental file 1 for details about training procedures). CDRS-R assessments will be videotaped to allow sample checking and for use in rating exercises for all preassessors and follow-up assessors each semester during the trial, to maintain and report inter-rater reliability for the primary outcome. The CDRS-R has good internal consistency and construct validity and is a good measure of symptom change.^42 43^

#### Secondary outcome measures

##### Assessor-rated measures

Secondary assessor-rated outcome measures are the *Children’s Global Assessment Scale*^44^ (total range 1–100, higher values represent higher functioning) and the *Clinical Global Impression-Severity Scale* and *Clinical Global Impression-Improvement Scale* (CGI-I)^45^ (total range 1–7, higher values indicate more severity/less improvement). In line with previous trials of MDD (eg, Treatment for Adolescents with Depression Study)^46^, treatment response is defined as a CGI-I rating of 1 or 2 (very much or much improved) at the 3-month follow-up compared with baseline. Further, the percentage of participants that still fulfil criteria for MDD diagnosis according to the DSM-5 criteria at the 3-month follow-up will also be presented.

Other assessor-rated measures are the Internet Intervention Patient Adherence Scale^47^ (I-BA groups only). Data on treatment content in TAU (eg, type, indication for medication, type of psychological treatment, number of visits) will be obtained from interviewing the families after the 3-month follow-up. Data on therapist time, primarily for giving feedback in the treatment platform, are logged manually. Phone call data will be presented as part of therapist time as well as separately.

##### Adolescent-rated and parent-rated measures

Depressive symptoms will be measured using the QIDS-17^48^ (total range 0–27, higher values indicate more severe depression). Irritability will be assessed with the *Affective Reactivity Index*^49^ (total range 0–12, higher values indicate worse outcome) and anhedonia by the *Anhedonia Scale for Adolescents*^50^ (total range 0–42, higher values indicate more anhedonia). Impaired functioning due to depression will be measured with the *Work and Social Adjustment Scale*^51^ (total range 0–40, higher values indicate greater impairment).^51^ *KIDSCREEN-10 Index*^52^ will be used to measure general health-related quality of life (total range 10–50, higher values indicate better quality of life). Anxiety symptoms will be assessed with the anxiety subscales (15 items) in the *Revised Children’s Anxiety and Depression Scale-Short version*^53^ (total range 0–45, higher values indicate greater severity of anxiety symptoms).^54^ Difficulties with sleep will be measured by the *Insomnia Severity Index* (total range 0–28, higher values indicate a worse outcome). The *Client Satisfaction Questionnaire* will be used to measure participants’ satisfaction with treatment^55^ (total range 8–32, higher values indicate higher satisfaction). Four qualitative questions will be administered to measure how credible participants perceive the treatments to be (total score 4–20, higher scores indicate more credibility). The *20-item Negative Effects Questionnaire* will be used to investigate participants’ negative effects of psychological treatments^56^ (total range 0–80, higher values represent more negative effects). To investigate whether the participant considers her/himself in need of further treatment for depression, we will use a scale that ranges from 0 (no need for more treatment) to 4 (great need for more treatment). Further, data will be collected on concurrent interventions, for example, about medication or psychological interventions in addition to what is included in the study arms. To track changes in activation and avoidance, the proposed mediators of BA, we will use the *Behavioral Activation of Depression Scale-Short form*^57^ (total range 0–54, higher values indicate a higher degree of activation and lower degree of avoidance). The *Expressed Emotion Adjective Checklist* will be used to assess the parent’s perceptions of positive and negative emotions towards the adolescent^58^ (total range 20–160, higher values represent more positive expressed emotions). The *Trimbos/iMTA questionnaire for Costs associated with Psychiatric illness* (TiC-P)^59^ will be administered to parents to measure resource use and other costs associated with psychiatric conditions. The TiC-P covers various healthcare costs (eg, visits to doctors, nurses or psychologist), medications, support and assistance (eg, study help), parental absence from work (eg, due to childcare) and productivity loss in school. Further, the adolescents will answer qualitative questions about other treatments they have received apart from trial interventions (ie, *co-occurring treatments*). This approach captures healthcare seeking by adolescents without their parents’ knowledge. While data from this measure will not be used in health economic evaluations, it will allow us to explore potential differences between parent and adolescent reporting on care-seeking behaviours.

### Patient and public involvement

During the development of the I-BA interventions, we involved patient representatives who had previously suffered from depression to provide feedback on language, and to ensure that the content was inclusive (eg, regarding sexual orientation and gender identity), clear and useful. We have also conducted a qualitative study^60^ in parallel with our feasibility study,^19^ which informed the improvement of the treatment content and appearance. For instance, we changed the appearance of one of our fictional characters and reduced the amount of text and follow-up questions in the modules.

### Power analysis

To estimate power, we conducted a simulation study with 2000 randomly generated datasets based on the information available from our feasibility study.^19^ In each generated dataset, we estimated a random intercept model with CDRS-R as outcome and time (numerical: postrandomisation months 0, 3 and 6), treatment (three-level categorical with TAU as reference group) and the interaction terms between month and treatment groups as covariates. We tested the null hypothesis that the interaction terms were jointly equal to zero with a 0.05-level Wald test. A sample size of 215 participants yielded 80% power to detect a statistically significant difference of 6 points in CDRS-R between each experimental group and the TAU group at the 3-month follow-up (6 months after randomisation). We based our power calculation on the difference in raw CDRS-R scores between groups, rather than on effect size alone. This approach, consistent with Cuijpers *et al*,^61^ recognises that effect size may not fully capture clinically significant differences. We deemed a 6-point difference on the CDRS-R clinically meaningful, based on influential trials in adolescent depression. While benchmarks vary—for example, Merry *et al*^62^ used a 5.5-point non-inferiority margin and Yoshimatsu *et al*^63^ suggest a 14-point change for minimal improvement—our 6-point choice is close to the 7-point threshold they consider relevant. This 6-point difference corresponds to a standardised mean difference of 0.5, a common threshold for superiority in clinical trials. Additionally, findings from the feasibility study^17^ suggest that an effect of this magnitude can be expected between each of the I-BA groups and TAU.

### Statistical analyses

#### Baseline data

Baseline sociodemographic and clinical data will be presented using descriptive statistics. In line with the Consolidated Standards of Reporting Trials 2019 statement,^64^ we will not perform significance testing of baseline differences between study arms.

#### Primary outcome analysis

Statistical analyses concerning clinical efficacy and 12-month durability will be conducted under the guidance of the Karolinska Institutet Biostatistics Core Facility (www.biostatcore.ki.se). Two linear mixed regression models will be used to estimate interaction effects between time and group for the primary outcome (the CDRS-R), between (1) therapist-guided I-BA versus TAU and (2) self-guided I-BA versus TAU. The models will include fixed effects for time and subject-specific effects as a random intercept factor to account for variances between and within participants. In contrast to standard modelling of repeated data, where listwise deletion is used for all cases with missing data at any time point,^65^ the linear mixed model estimates effects using all available observations at all time points, that is, according to the intention-to-treat principle. Linear mixed models yield reliable estimates in various types of missing data scenarios.^66^ The estimated interaction effect will be reported with an accompanying 95% CI and p value. The alpha level throughout this trial will be set to p<0.05 as a threshold for statistical significance. Between-group effect sizes (Cohen’s d) will be calculated using the accumulated beta coefficients (pretreatment to 3-month follow-up) from the regression models as the nominator and the pooled SD at pretreatment as the denominator.^67^

#### Secondary outcome analyses

Secondary outcomes will be analysed using a similar statistical approach as the primary outcome, that is, with linear mixed models. The results will be presented as estimates with their respective 95% CIs and p values. Dichotomous variables will be analysed using logistic mixed models. Additionally, the proportion of treatment responders at 3-month follow-up will be calculated with completers data according to the prespecified criteria.

#### Long-term follow-up analyses

For the long-term follow-up, analyses will also be conducted using a similar approach as for the primary outcome. The regression models will include the 3 and 12-month follow-up assessment points and evaluate whether the potential short-term treatment effects in each intervention group are maintained at the 12-month follow-up. In addition, we will also enter all available assessment points into a separate regression model to examine whether there are significant interaction effects at the 12-month follow-up.

#### Health economic evaluations

Cost data will be analysed from three perspectives: healthcare provider, healthcare system and societal perspective. Each perspective will undergo cost-utility analysis (costs in relation to quality-adjusted life years (QALYs)) and cost-effectiveness analysis (costs in relation to treatment effects, using treatment response as the clinical outcome). Individual participant resource use frequencies at baseline, post-treatment and 3-month follow-up will be multiplied by unit costs. Costs will be converted from Swedish krona to euros based on annual conversion rates. KIDSCREEN-10 scores will be converted to QALYs using established algorithms.^68^ Between-group cost differences at the primary endpoint and 12-month follow-up will be analysed using generalised linear models. Results will be presented as incremental cost-effectiveness ratios, the ratio of cost and effect differences between the interventions, indicating the additional costs or savings for one additional QALY or participant in remission.

Non-parametric bootstrapping with 5000 repetitions will be used for calculating means and 95% CIs due to the expected skewed nature of cost data. Results will be visualised in cost-effectiveness planes, displaying the probability distributions of relative cost savings in relation to gains in QALYs and proportion of treatment responders. Sensitivity analyses, assessing result robustness, involve calculating the probability of cost-effectiveness across various willingness-to-pay scenarios (cost-effectiveness acceptability curve), and by increasing clinician costs by 50%. Analyses will be repeated for each contrast of comparators: therapist-guided I-BA versus TAU and self-guided I-BA versus TAU.

### Quality control

The trial will be conducted according to Good Clinical Practice (GCP) standards. An introductory course in GCP is mandatory for all trial staff. GCP documents such as source data, task delegation lists and deviation log will be established. All quality and safety aspects will be regularly monitored by KTA (ie, case-by-case monitoring of informed consent, eligibility criteria, source data quality and serious adverse events). Approximately 160 hours are estimated for monitoring during the trial.

### Ethics and dissemination

This study is conducted according to the Declaration of Helsinki^69^ and GCP. The Swedish Ethical Review Authority has approved this study (ref number: 2021-02555 with amendments: 2022-01847-02; 2022-04582-02; 2023-05398-02; 2023-07036-02). Written informed consent will be obtained from all participants and parents prior to the inclusion assessment. All participating families will be volunteers, competent to give informed consent. Participants can withdraw from the trial at any time. The results of this study will be submitted for publication in peer-reviewed international journals, presented at scientific conferences and communicated to healthcare providers and the public.

### Trial status

Recruitment started on 6 September 2021 and ended at the beginning of May 2024. The last participant is expected to reach the primary endpoint in November 2024. The controlled 1-year follow-up will continue until the autumn of 2025. No interim analyses are planned, thus results on outcomes will not inform decisions to stop the study. Any serious adverse events, however, will be reviewed, and if there is any indication that these are linked to the intervention, consideration will be given to discontinuing the trial on the advice of the trial sponsor and participating clinical services. Failure to recruit could also be a reason to stop the trial.

## supplementary material

10.1136/bmjopen-2023-083507 online supplemental file 1

10.1136/bmjopen-2023-083507 online supplemental file 2
