# Supplementary material for: Single-blinded, randomised, parallel-group, controlled trial comparing the efficacy and cost-effectiveness of therapist- and self-guided internet-delivered behavioural activation versus treatment as usual for adolescents with mild to moderate depression: study protocol
Source: BMJ Open. 2024 Oct 15;14(10):e083507. doi: 10.1136/bmjopen-2023-083507 (PMC11590840; doi:10.1136/bmjopen-2023-083507)
Supplement: online supplemental file 1 [file bmjopen-14-10-s001.pdf]

STUDY PROTOCOL IDA RCT

Long title of the trial	Efficacy and cost-effectiveness of internet-delivered behavioural activation for adolescents with mild to moderate depression: A randomised controlled trial of guided and self-guided I-BA vs. treatment as usual
Short title of trial	The IDA trial
Version and date of protocol	Version 1.5., 2023-10-27
Sponsor	Barn- och ungdomspsykiatrin (BUP) Stockholm, Region Stockholm
Funders	Kavli Trust; Frimurare Barnhuset in Stockholm and Lund; Child and Adolescent Mental Health Care Services Lund; ALF Medicin, Region Stockholm; the Swedish Research Council; Jane and Dan Foundations; Stiftelsen Konung Oscar II:s and Drottning Sophias Guldbröllopsminne
Trial registration number	ClinicalTrials.gov: NCT04977856
Sites	Child and Adolescent Psychiatry Research Center, Gävlegatan 22, Stockholm, Sweden
Principal investigator	Professor Eva Serlachius: eva.serlachius@ki.se Department of Clinical Neuroscience (CNS)
Sponsor representative	Göran Rydén, MD, Head of Child and Adolescent Mental Health Services, Region Stockholm

VERSION HISTORY

Version number	Version date	Reason for change
1.0	2021-04-15	First version
1.1	2021-08-31	- Section 5.7.4 "Secondary adolescent-reported outcomes", a description of the measure "Treatment credibility" for adolescents was added since it was lacking.<br>- Page 1, clinicaltrials.gov identifier and changed from adjunct professor to professor for Eva Serlachius.<br>- Page 22, changed sentence "Before: ... verify (depression?) diagnosis according to MINI-KID, (...) assess current severity level of depression (CDRS-R)." to "At the diagnostic assessment visit, a licensed psychologist will a) verify major depressive disorder diagnosis according to DSM-V, b) assess psychiatric comorbidity, c) decide on inclusion/exclusion and d) assess current severity level of depression, and ask about relevant medical history.<br>- Page 34, added definitions of grading scale of AE/SAE.<br>- Section 6.2, clarified procedures for collecting informed consent.<br>- Section 6.3 and 6.5, added time-windows for data collection.<br>- Added section 6.7. about quality control with details on monitoring of data quality and safety.<br>- Added information on handling and assessment of AE/SAE to section 9.3 A-C.<br>- A description of training in the primary outcome measure was added to section 5.7.2<br>- Page 30 in table 2, added parents to "Demographic data" since it unintentionally had been left out<br>- Page 36, removed "on a specific SAE-form" from this sentence: "SAEs will be recorded in the patient's medical record (Take Care), on a specific SAE-form, as well as in the trial coordinator's log", since we are not familiar of a specific form in the medical journal.<br>- Page 36, section 9.4. removed that NEQ will be reported as AE, since it will be separately reported in manuscript.<br>- Page 3, added space for signature from sponsor representative.

1.2.	2021-09-07<br><br><br><br><br>2021-09-22<br><br>2021-09-22	- Page 35, About assessment of causal relationship with the intervention, the part marked in bold has been added: "The final decision is taken by the PI, or if she is not present, by team member and medical doctor Anna Ohlis.<br>- Page 18, minor changes in procedures for training of CDRS-R (step 5). Please see Note to file 7.<br>- Page 25, details on randomisation service has been added.
1.3.	2022-03-25	- Updated section 6: (a) To improve inclusion rate, from Autumn 2022 patients will also be recruited directly from BUP Lund; (b) Written consents will be collected on paper or digitally through a secure platform.
1.4.	2022-08-19<br><br>2022-08-22	- We change from Zoom to Teams for video-meetings. See 7.1.2., p 33.<br>- We will use information from medical records from BUP Lund to make our initial assessment more efficient. See 6.2., p 23.
1.5.	2023-10-27	- Reimbursement to participants at completed three-month follow-up has been added; See 6.5.3 Follow-up-assessments, p 29.<br>- Updated power calculation due to a miscommunication with the trial statistician in the original power calculation. See 5.1 Design p 13; 5.4 Power analysis, o 14.Updated details on funders, See table, p 1.

SIGNATURES

Protocol version number: 1.5.
Eva Serlachius, Principal Investigator (PI)
Date: 231102
Signature: Eva Serlachius

Please see an electronic signature at the end of the document.

Protocol version number: 1.5.
Göran Rydén, sponsor representative
Date: 231110
Signature: [illegible]

TABLE OF CONTENTS

1 TRIAL PERSONNEL 6
2 SUMMARY 8
3 BACKGROUND AND RATIONALE 11
4 OBJECTIVES 13
4.1 PRIMARY AND SECONDARY OBJECTIVES 13
5 PROJECT DESCRIPTION 13
5.1 DESIGN 13
5.2 CONTROL GROUP JUSTIFICATION 14
5.3 STUDY SETTING 14
5.4 POWER ANALYSIS 14
5.5 ELIGIBILITY CRITERIA 15
5.5.1 Inclusion criteria 15
5.5.2 Exclusion criteria 15
5.6 DATA COLLECTION 16
5.7 MEASURES 16
5.7.1 BASELINE MEASURES 16
5.7.2 PRIMARY OUTCOME 17
5.7.3 SECONDARY CLINICIAN-RATED OUTCOMES 18
5.7.4 SECONDARY ADOLESCENT-REPORTED OUTCOMES 19
5.7.5 SECONDARY CAREGIVER -REPORTED OUTCOMES 21
6 PROCEDURES 23
6.1 RECRUITMENT RATE 23
6.2 SCREENING AND RECRUITMENT PROCEDURES 23
6.3 RANDOMISATION, ENROLMENT AND MASKING 25
6.4 TREATMENT 26
6.4.1 GUIDED AND SELF-GUIDED I-BA 26
6.4.2 TREATMENT AS USUAL (TAU) 28
6.4.3 THERAPIST TRAINING, SUPERVISION AND MONITORING 29
6.5 SUBSEQUENT ASSESSMENTS 29
6.5.1 ASSESSMENTS DURING TREATMENT 29
6.5.2 POST-TREATMENT ASSESSMENT 29
6.5.3 FOLLOW-UP ASSESSMENTS 29

6.6 END OF TRIAL 31
6.6.1 PARTICIPANT WITHDRAWAL FROM TRIAL 31
6.6.2 DISCONTINUATION OF TRIAL 31
6.6.3 CONCOMITANT INTERVENTIONS 31
6.7. QUALITY CONTROL 31
7 DATA MANAGEMENT 32
7.1 DATA COLLECTION AND HANDLING 32
7.1.1 BASS 32
7.1.2 Teams 33
8 STATISTICAL ANALYSIS 33
9 RECORDING AND REPORTING OF ADVERSE EVENTS AND REACTIONS 34
9.1 DEFINITIONS 34
9.2 EXPECTED ADVERSE EVENTS 34
9.3 ASSESSMENT OF ADVERSE EVENTS 35
9.4 HANDLING OF ADVERSE EVENTS 36
10 PUBLIC AND PATIENT INVOLVEMENT 36
11 DATA SHARING 37
12 ETHICAL CONSIDERATIONS 37
13 IMPLICATIONS 38
14 FINANCE 39
15 PUBLICATION PLANS 39
16 REFERENCES 39

1 TRIAL PERSONNEL

Principal Investigator (PI): Professor Eva Serlachius
Karolinska Institutet
Department of Clinical Neuroscience (CNS), K8,
Gävlegatan 22, Plan 8 113 30 Stockholm, Sweden
eva.serlachius@ki.se
+46 70-715 52 32

Sponsor representative: Göran Rydén
Barn- och ungdomspsykiatrin
Region Stockholm
Sachsgatan 10, 118 61 Stockholm
goran.ryden@sll.se
+46 8-514 520 88

Co-investigators: Dr Sarah Vigerland
Karolinska Institutet
Department of Clinical Neuroscience (CNS), K8,
Gävlegatan 22, Plan 8 113 30 Stockholm, Sweden
sarah.vigerland@ki.se
+46 76-172 16 09

Dr Johan Åhlén
Karolinska Institutet
Department of the Department of Global Public Health
johan.ahlen@ki.se

Professor David Mataix-Cols
Karolinska Institutet
Department of Clinical Neuroscience (CNS), K8,
Gävlegatan 22, Plan 8, 113 30 Stockholm, Sverige
david.mataix.cols@ki.se
+46 72-398 68 89

Dr Fabian Lenhard
Karolinska Institutet
Department of Clinical Neuroscience (CNS), K8,
Gävlegatan 22, Plan 8 113 30 Stockholm, Sweden
fabian.lenhard@ki.se

PhD-student Rebecca Grudin
Karolinska Institutet
Department of Clinical Neuroscience (CNS), K8,
Gävlegatan 22, Plan 8 113 30 Stockholm, Sweden
rebecca.grudin@ki.se
+46 70 261 59 68

2 SUMMARY

Title: Efficacy and cost-effectiveness of internet-delivered behavioural activation for adolescents with mild to moderate depression: A randomised controlled trial of guided and self-guided I-BA vs. treatment as usual

Short title: Internet-delivered psychological treatment for adolescents with depression

Aim and objectives: The overall aim of this research project is to increase the availability of evidence-based psychological treatments for adolescents with depression by developing and evaluating internet-delivered Behavioural activation, I-BA) for this target group. The main objectives are to establish the efficacy, cost-effectiveness and long-term effects of the I-BA intervention in adolescents with mild to moderate MDD in an RCT with three-arms; guided I-BA (with therapist-support) vs self-guided I-BA (without therapist-support) vs treatment as usual (TAU).

Type of trial: Single-blind parallel-group randomised controlled trial

Trial design and methods: All potential participants are initially screened via telephone. This is followed by an inclusion assessment conducted at the clinic. Participants who are eligible and have consented will be randomised to one of three trial arms. In the experimental arms, participants receive either 10 weeks of guided or self-guided internet-delivered behavioral activation (BA). In the control arm, participants receive TAU within the Child and Adolescent Mental Health Services (CAMHS) or primary care clinics.

Participants will complete outcome measures at baseline, mid-treatment, post-treatment, at 3 months and 12-months follow-up assessments. Depressive symptoms and suicidality will be monitored weekly. The primary clinical outcome variable is clinician-rated depressive symptoms (CDRS-R), and the primary endpoint is the follow-up 3-months post-treatment.

	Post- and follow-up assessment will be conducted at the clinic or via telephone or video, both times complemented with online questionnaires.
**Rationale for study:**	Adolescent depression is a prevalent and impairing condition that can be effectively treated with Cognitive Behavior Therapy (CBT). However, a majority of adolescents do not have access to CBT. Internet-delivered CBT (ICBT) has been suggested as a way to increase availability to effective psychological treatments. Yet, the research on ICBT for adolescents has been lagging behind significantly when it comes to quantitative.
**Participant time in trial:**	Approximately 15 months (follow-up included).
**Total trial duration:**	Approximately 34-months (from first participant enrolled to last participant follow-up).
**Planned trial sites:**	All assessments and delivery of treatment will be administered from a single site in Stockholm, the CAP Research Centre. Regions all across Sweden will be able to refer potential participants to the Stockholm site for inclusion in the trial as well as delivering the TAU condition.
**Sample:**	215 participants allocated to one of the 3 treatment arms at a ratio of 1:1:1 (75 in each group).
**Brief eligibility criteria:**	Eligible participants will be aged 13-17 years and have a DSM-5 diagnosis of mild or moderate major depressive disorder (MDD). Exclusion criteria include acute problems such as severe MDD, suicidal risk, other primary psychiatric disorder, ongoing maltreatment or abuse, receiving CBT within the past 12 months, and recent changes in psychotropic medication. Full details are presented in section 5.5.
**Statistical analysis:**	*Demographic data* will be summarized using descriptive statistics.

Efficacy. Analyzing and reporting of data will be conducted according to *CONSORT* guidelines. Analyses of continuous measures (primary and secondary outcomes) are conducted according to the "intent-to-treat" principle, including all randomised participants in hierarchical linear mixed-models analysis. Linear mixed model is also an adequate method to handle missing data. Descriptive statistics on withdrawal, drop-out, and attrition rates, treatment satisfaction, treatment credibility and therapist time will be presented.
Cost-effectiveness. Cost data will include resource use costs (e.g., hospital visits, medication and absence from school, caregiver's absence from work) and cost of I-BA or TAU. Cost differences between guided I-BA, self-guided I-BA and TAU can be calculated, as well as the incremental cost effectiveness ratio (ICER). The ICER is a global estimate of cost-effectiveness and represents the additional cost that is associated with the additional clinical effect in one intervention compared with another.

3 BACKGROUND AND RATIONALE

Depression is a psychiatric disorder characterized by multiple and persistent behavioral, cognitive and physical symptoms, such as feeling of sadness and hopelessness, irritability and a feeling that life is no longer worth living. People affected by depression commonly lose interest or pleasure in activities that they used to enjoy. Changes in sleep, appetite, and cognition, and thoughts about death are often experienced. Diagnostic criteria are met when depressive symptoms have been present during a 2-week episode and when function in everyday life is impaired (1).

MDD has a large impact on mortality, disability and quality of life (2). The World Health Organization identifies major depressive disorder as a leading cause of burden of disease and a global health priority (3). Adolescence is a vulnerable period for developing MDD, involving a sharp increase in prevalence rates especially for girls (4). By the end of adolescence, approximately 10 % have experienced an episode of MDD (5). Adolescent MDD is associated with significant secondary problems (6) and strongly predicts mental and somatic health problems, as well as psycho-social problems in adulthood (7, 8). Importantly, early detection and treatment of adolescent MDD markedly decreases the likelihood of future depressive symptoms (9).

Cognitive behavioral therapy (CBT) is recommended as a first line treatment for adolescents with mild to moderate MDD (10). One type of CBT for depression is behavioral activation (BA). BA is based on the behavioral theory of depression and aims to improve mood by increasing positive reinforcement for healthy behaviors. Unlike other forms of CBT, BA does not include cognitive components. According to a meta-analysis by Cuijpers and colleagues (11) BA is equivalent to other forms of CBT for adults with depression, and can be more effective than medication (12). Furthermore, Cuijpers et al (11) concluded that BA was the most successful treatment in regard to percentage of participants no longer meeting criteria for MDD after treatment. Dismantling studies have suggested BA as a promising candidate of being a sufficient treatment component in its own for depression (13, 14). BA has the advantage of being uncomplicated, time-efficient and not requiring complex skills from either participants or therapists. Many young people across all types of psychological treatments drop out prematurely (15). BA, being brief and simple to understand, might therefore fit adolescents especially well.

Although effective interventions exist, only a small percentage of patients with depression get access to these (16). Epidemiological studies reveal significant underutilization of mental health care. For example, Essau (17), showed that only 23 % of adolescents with depressive disorders got access to mental health services.

Over the last decade, internet-delivered cognitive behavior therapy (ICBT) has been developed for several different psychiatric conditions. This makes it possible to offer less

therapist-intensive, but effective, interventions over long distances, which can increase the availability of evidence-based treatments. In ICBT the participant follows an interactive online treatment manual that mirrors the content, components and strategies from standard face-to-face CBT, either guided or self-guided (i.e., with or without therapist-support). Crucially, ICBT only requires a fraction of the therapist time associated with regular CBT (18).

In a review of 108 randomised controlled studies (RCTs), ICBT was found to be effective and probably cost-effective for several adult psychiatric disorders (19). Furthermore, ICBT is at least as efficacious as face-to-face CBT for a number of adult psychiatric disorders, including MDD (20). Comparatively less research has been conducted on ICBT for adolescent depression. To our knowledge only four studies have evaluated if ICBT is effective for young people with depression. In the first with a sample of 38, no significant effects of ICBT on depressive symptoms were found(21), whereas the other study, with a larger sample, found a moderate effect of self-guided ICBT compared to an educative control condition(22). However, both studies suffered from severe methodological shortcomings, such as small sample sizes and only including individuals with subthreshold, rather than diagnosed depression. In the third and fourth studies promising results were shown for ICBT with additional therapist-chat communication, i.e., blended ICBT, for adolescents aged 15 to 19 years (n=70 in both) with diagnosed depression compared with attention control(23) and minimal attention control(24). One limitation of study three and four was that comparison between groups was not possible at 6-months follow-up due to the cross-over design. Also, at post treatment assessors were not blinded to participant allocation. Furthermore, the use of attention control, as compared to a more active control condition, might have overestimated the results. Blended formats do not have the same potential advantage of saving therapist time compared to traditional CBT. Other studies focusing on internet-delivered psychological treatment and adolescent depression have mainly been prevention trials (25) or have used computerized programs that have not utilized the possibility to overcome geographical distances (26).

The limited research available leaves many questions unanswered. First, there is a need for well-designed, methodologically sound and properly powered trials in the field. Second, whether therapist support is important in ICBT for adolescent depression is a critical question. If ICBT could be entirely self-guided, without sacrificing efficacy and safety, it would be much easier to disseminate. Third, the cost-effectiveness of ICBT for adolescent MDD has not been evaluated. Lastly, it is unknown whether the beneficial effects of ICBT for adolescent MDD are durable in the long run.

In October 2020 we completed a pilot study on I-BA (a version of an ICBT intervention) with N=32. This study showed that the study design is feasible in terms of recruitment rate,

treatment acceptability and patient-safety. We observed symptom reduction on depressive symptoms (the clinician-reported Children's
Depression Rating Scale, Revised (primary outcome) with large effects in all three groups from pre to 3-months follow-up, with larger effects for guided and unguided I-BA than TAU, with significant within-group results for the I-BA groups only.

4 OBJECTIVES

The overall goal of our research project is to increase the availability to evidence-based psychological treatments of adolescent MDD.

4.1 PRIMARY AND SECONDARY OBJECTIVES

Primary:

1. To determine the clinical efficacy of Guided and Self-guided I-BA for depression for reducing depressive symptom severity (as measured by the CDRS-R (27) in adolescents with mild and moderate MDD, compared with an active control intervention (treatment as usual within primary or secondary child and adolescent mental health care). The primary endpoint is the follow-up 3 months post-treatment.

Secondary:

1. To establish the 12-month durability of the treatment effects.
2. To conduct a health-economic evaluation of guided I-BA for depression and self-guided I-BA for depression, compared with TAU, from multiple perspectives, both in the short term (primary endpoint) and the long term (12-month follow-up).

Research questions:

1. Is guided and self-guided I-BA more efficacious than TAU in regard to reduction of depressive symptoms?
2. Is guided and self-guided I-BA more cost-effective than TAU?
3. Is self-guided I-BA more cost-effective than guided I-BA?
4. Are the therapeutic gains of I-BA maintained long term (i.e., 1 year after the intervention)?

5 PROJECT DESCRIPTION

5.1 DESIGN

This will be a single site randomised controlled trial with national recruitment that will include 215 adolescents with mild to moderate depression. Participants will be randomised to receive 10 weeks of either (1) guided I-BA or (2) self-guided I-BA or (3) treatment as usual (TAU), at a ratio of 1:1:1 (72 in each group). TAU will be provided by regular mental health care clinics, within either primary or secondary care.

5.2 CONTROL GROUP JUSTIFICATION

The choice of type of control condition in a trial for a psychological intervention depends on several factors including the disease and its characteristics, the development phase, the purpose of the study, and the resources available (28). We decided on treatment as usual for the following reasons. (1) We are interested in comparing I-B to TAU, to see if it is less, equally or more effective than TAU. We are also interested in evaluating if it is cost-effective compared to TAU. (2) We consider depression (mild to moderate) to be associated with an intermediate patient risk and that contact with health care is necessary to ensure patient safety. (3) Waitlist would not be the most ethical choice since there are more efficient treatment options available for depression. (3) Face-to-face CBT could be an option to TAU, but demands more resources. It is also a more rigid control condition which involves a risk of underestimating the effect of I-B. Since this is an early-stage trial of a rather novel format for delivering CBT for depression, where we hope to evaluate whether or not the intervention is effective, we believe TAU is more appropriate. (4)

5.3 STUDY SETTING

The study will be conducted at the Child and Adolescent Psychiatry (CAP) Research Centre within Child and Adolescent Mental Health Services (CAMHS) in Stockholm in collaboration with the Department of Clinical Neuroscience at Karolinska Institutet. Participants will formally be considered as patients at the CAP Research center from enrolment to the 3-month follow-up (primary endpoint). All assessments will be administered from a single site, the CAP Research center, at Gävlegatan 22 in Stockholm. All counties across Sweden will be able to refer potential participants to the Stockholm site for inclusion in the trial and will be responsible for delivering TAU if participants from their county is randomised to the control group.

5.4 POWER ANALYSIS

The sample size has been calculated with the help of KI Biostatistics Core Facility (www.biostatcore.ki.se). To estimate power, we conducted a simulation study with 2,000 randomly generated datasets based on the information available from our feasibility study (Grudin et al., 2022). In each generated dataset, we estimated a random intercept model with CDRS-R as outcome and time (numeric: post-randomisation months 0, 3 and 6), treatment (three-level categorical with TAU as reference group), and the interaction terms between month and treatment groups as covariates. We assumed a data availability of 85% at 3 months and 77% at 6 months. We tested the null hypothesis that the interaction terms were jointly equal to zero with a 0.05-level Wald test. A sample size of 215 participants yielded 80% power to detect a statistically significant difference of six points in CDRS-R between each experimental group and the TAU group at the 3-month follow-up (6 months post-randomisation). Six points correspond to a medium standardized effect size of 0.5 and align well with our clinical impression of minimal clinically meaningful change. Additionally, findings from the feasibility study (Grudin et al., 2022) suggest that an effect of this magnitude can be expected between each of the I-BA groups and TAU.

This power calculation was updated in September 2023. See Appendix 1 for detailed information.

5.5 ELIGIBILITY CRITERIA

5.5.1 INCLUSION CRITERIA

- 13-17 years of age,
  - Confirmed by the caregiver and subsequently by the medical records.
- A principal diagnosis of mild or moderate MDD as defined by the 5th edition of the Diagnostic and Statistical Manual of Mental Disorders(1).
  - Confirmed by the assessor at the inclusion assessment with the help of a semi-structured clinical interview, clinician-rated depression interview and clinician assessment of global functioning.
- Willing to be randomised to either of the three treatment arms.
  - Confirmed by the assessor at the inclusion assessment.
- Basic proficiency in Swedish, both adolescent and participating caregiver
  - Confirmed by the assessor at the inclusion assessment.
- Regular access to a desktop or laptop computer connected to the internet, as well as a mobile phone to receive SMS (one of each is enough per family).
  - Confirmed by the caregiver at the telephone screening or/and inclusion assessment.
- If using medication with antidepressants, central stimulants and neuroleptics it has to be unchanged at least 6 weeks prior to inclusion.
  - Confirmed by the assessor at the inclusion assessment. Also followed up at post and follow-up assessment point in the trial.
- A minimum of one caregiver that is able to co-participate in the treatment
  - Confirmed by the caregiver at the telephone screening or/and inclusion assessment.

5.5.2 EXCLUSION CRITERIA

1) Immediate risk to self or others, requiring urgent medical attention, such as suicidality, or repeated self-injurious behavior.
   - Confirmed by the assessor at the telephone screening or/and face-to-face inclusion assessment, with the help of a structured clinical interview and a screening instrument for assessing non-suicidal self-harm inventory (DSHI-7).
2) Other psychiatric disorders or social problems requiring other actions at first hand (e.g., diagnosis of organic brain disorder, intellectual disability, psychosis, bipolar disorder, eating disorder, or alcohol/substance dependence; ongoing bullying in school, abuse and/or neglect in the family; high and prolonged absence from school).

- Confirmed by the assessor at the telephone screening or/and face-to-face inclusion assessment, with help of the clinical interview and information from the caregiver and the adolescent.

3) Previous psychological treatment for MDD for a minimum of at least 3 sessions within the last 12 months prior to assessment.
    - Confirmed by the caregiver at the telephone screening or/and face-to-face or inclusion assessment.
4) Current use of benzodiazepines.
    - Confirmed by the caregiver at the telephone screening or/and face-to-face or inclusion assessment.
5) Ongoing psychological treatment for any other psychiatric disorder.
    - Confirmed by the caregiver at the telephone screening and/or face-to-face inclusion assessment.

Excluded families will be directed to other more suitable treatment options.

5.6 DATA COLLECTION

Clinician assessments, based on semi-structured interviews with both the adolescent and the caregiver, will be conducted at the clinic or through video calls. Post- and follow-up interviews can be conducted over video if a meeting at the clinic is not possible and to minimize data loss. The measures used are validated, reliable, and routinely employed in depression and child psychopathology trials. All assessors will be trained by experienced clinicians. The semi-structured interview (the Children's Depression Rating Scale-Revised) will be audio-recorded to explore inter-rater reliability between different assessors. Self- and caregiver-reported measures are collected through an online platform where no missing single items are allowed.

5.7 MEASURES

Clinician-rated/administered measures will first be recorded on paper, then entered into the online trial database, called BASS. Adolescent and caregiver-reported measures will be completed online and entered directly into BASS. More information about BASS can be found in section 7.1.1. For the specific time points of administration for each measure, see section 6.5, Table 2.

5.7.1 BASELINE MEASURES

Demographic data – clinician-entered

Following the face-to-face inclusion assessment, the assessor enters baseline information into the trial database. This includes information on screening number, age, sex, primary responsible caregiver assisting during the treatment, depression diagnosis, comorbid diagnoses, whether they live in a rural or urban area, age of onset for depression, previous episodes of depression, history of suicide attempts, current medication for depression

disorder or other psychiatric disorder and previous psychological treatment for depressive disorder.

Demographic data – caregiver-reported

At baseline, the caregiver will answer a questionnaire designed by the research team, asking about the caregiver's relation to the child, who the child lives with, if the child has any siblings, the caregivers' educational level, the caregivers' occupation, where the child and caregivers are born, how the caregiver learned about the trial, if the child has been in previous contact with health care services due to her/his depression, or other psychiatric symptoms.

Mini International Neuropsychiatric Interview for Children and Adolescents (MINI-KID)

The MINI-KID (29) is a short semi-structured diagnostic interview for DSM-IV and ICD-10 psychiatric disorders in children and adolescents. It will be used to assess psychiatric comorbidity at baseline and may be used to exclude participants. All clinicians will receive training in MINI-KID prior to the trial. Inter-rater and test-retest reliability coefficients have been shown to be acceptable to excellent. MINI-KID has been validated against the widely used K-SADS-PL where good concordance was found for mood disorders (29).

The Deliberate Self Harm Inventory for youths – (DSHI-Y-7)

The 7-item DSHI for youths that will be used to assess presence and frequency of non-suicidal self-injury, to help the clinician decide on inclusion/exclusion. The DSHI-Y-7 is adapted for adolescents from the DSHI-17 (30), an empirically supported measure of various aspects of NSSI originally developed for use with adults. The DSHI-17 has demonstrated good test-retest reliability and adequate concurrent validity among adolescents (30).

5.7.2 PRIMARY OUTCOME

Children's Depression Rating Scale, Revised (CDRS-R)

CDRS-R (27) is the most widely used rating scale for assessing severity of depression and change in depressive symptoms for clinical trials with children and adolescents (27, 31, 32). The CDRS-R is a semi-structured interview-based measure modelled on the adult Hamilton Rating Scale for Depression. The CDRS-R has been found to be a sensitive and reliable severity measure of depression in youth. Item values range from 1 to 5 or 1 to 7 with a possible total score from 17 to 113, with higher scores reflecting more clinically significant difficulties. A raw score of ≥ 40 is indicative of depression, while a score of ≤28 is often used to define remission (minimal or no symptoms, (32). CDRS-R has shown good internal consistency and good construct validity and is also considered a good measure of symptom change (32). Independent clinical evaluators (ICE's) will administer the 17-item scale to the adolescent. The CDRS-R can be completed in 30 to 45 minutes by an experienced assessor.

We will take extensive steps to minimize measurement bias of the primary outcome. All raters of CDRS-R will be trained according to the procedures below. The training will be supervised by a clinical expert, the trial coordinator Rebecca Grudin.

1) Training will consist of attending a short lecture by RG, where the CDRS-R is explained.
2) Raters will then listen to at least two pre-recorded CDRS-R assessments. They will be asked to rate these; however, this will be solely for training purposes. Raters are encouraged to ask questions. The questions and ratings will then be discussed with RG. The next step will be a testing phase where 3 different CDRS-R assessments will be used against an expert rater (RG) to determine the extent of agreement. In line with the methodology reported by Jeon et al. (33), the raters have to be within 15% of the expert rater for the total score on CDRS-R on each of the 3 recordings. The 15% will always be rounded up in cases where 15% of a score results in a score which is not a whole integer (i.e., 15% of 25 = 3.75 points, this would be rounded up to 4 points). The 15% can be in either direction of the score.
3) Raters who do not meet the criteria will be given additional training and asked to re-score the same CDRS-R assessments until the specified agreement criteria are met.
4) Once per semester during the trial all assessors will gather to make an assessment of a pre-recorded CDRS-R interview to discuss possible differences in ratings of individual items.

We will ask the participants' permission to video record all CDRS-R assessments for possible spot checks of the methodology and to measure inter-rater reliability.

5.7.3 SECONDARY CLINICIAN-RATED OUTCOMES

Clinical Global Impression Scale – Severity (CGI-S)

The CGI-S (34) is a single item clinician rating of symptom severity, in this trial used as an overall rating of the depression disorder severity. Ratings are made on a seven-point scale range from 1 ("no symptoms") to 7 ("extreme symptoms"). CGI correlates well with established outcomes scales such (35).

Clinical Global Impression Scale – Improvement (CGI-I)

The CGI-I (34) provides a clinician-rated opinion of global improvement. The measure consists of one single item, asking about the level of improvement compared to admission, which is rated according to the following: 1=very much improved; 2=much improved; 3=minimally improved; 4=no change from baseline (the initiation of treatment); 5=minimally worse; 6=much worse; 7=very much worse". The questionnaire has established validity and reliability (35). All assessors will practice rating CGI-I together and inter-rater reliability will be calculated. Treatment responders will be defined as scores of 1 (very much improved) or 2 (much improved) at the primary endpoint (36).

Children's Global Assessment Scale (CGAS)

The CGAS (37) is a single item 1-100 scale that integrates psychological, social, and academic

functioning in children as a measure of global functioning. The questionnaire is assessor-rated and has established validity and reliability (38).

Internet Intervention Patient Adherence Scale (iiPAS)

The iiPAS is a clinician-rated measure of patient adherence to internet-delivered behavioral interventions (39) with 5 items rated on a 0 to 4 Likert scale with total score ranging from 0 to 20, where 0 indicates no adherence and 20 perfect adherences. The scale covers client's work pace, engagement, communication with the therapist, motivation for change, and login frequency. The iiPAS has demonstrated excellent internal consistency and good construct validity as well as a strong association with objective measures of patient activity in ICBT(39). For participants in the self-guided group, we will use an adapted version excluding item 3 since communication with therapist is not applicable, and a research assistant will make the rating at post-treatment.

Treatment completion + therapist time

Usage data from the BASS platform will be extracted to measure completed modules for adolescents and their caregivers respectively, as well as total therapist time per family and week.

5.7.4 SECONDARY ADOLESCENT-REPORTED OUTCOMES

Anhedonia Scale for Adolescents (ASA)

The ASA (40) is an adolescent specific measure of anhedonia with 14 items with a four graded scale from 0 (never) to 3 (always), ranging from 0 to 42 points. A higher score indicates more anhedonia. The ASA has high test-retest reliability and good convergent validity with standardized measures of depression (40).

Affective Reactivity Index (ARI)

The ARI (41) is measure of irritability, consisting of six items with a three graded-scale and one item on impairment due to irritability, ranging from 0 to 12 points. The ARI has demonstrated an excellent internal consistency and differentiates cases from controls in a clinic a community sample (41).

Behavioral activation of Depression Scale- short form (BADS-SF)

The BADS-SF (42) is a 9 self-report measure designed to track changes in proposed mediators of BA: activation and avoidance. The item is scored on a scale from 0 (not at all) to 6 (completely). The BADS-SF has two subscales, activation (focused, goal-directed activation and completion of scheduled activities) and avoidance/rumination (avoidance of negative aversive states and engaging in rumination rather than active problem solving). The BADS-SF has acceptable internal consistency reliability, construct and predictive validity (42).

Concomitant interventions

To assess if the adolescent has received other psychological treatments during the trial period, the adolescents answer questions about psychological treatment (type, number of sessions, indication and time period) at post-treatment and at follow-up assessments.

Client Satisfaction Questionnaire (CSQ)

The CSQ (43) is an 8-item self-rated on a 4-point scale measuring different aspects of satisfaction with treatment, e.g., perception of quality of treatment, if the treatment adequately addressed their needs and overall satisfaction. The scale has high internal consistency and correlates with therapists' estimates of client satisfaction(43).

Insomnia Severity Index (ISI)

The ISI is a brief screening measure of insomnia, a seven-item scale ranging from 0 to 28 points. The scale is reliable and sensitive to change (44).

KIDSCREEN-10 Index

The KIDSCREEN-10 Index (45), consists of 10 items, each with a 5-level response category and an additional question on general health. This scale was developed from the longer KIDSCREEN-52 and is considered a valid measure to assess the adolescents' general health-related quality of life.

Need for further treatment

This is a single-item questionnaire created by the research team led by Professor David Mataix-Cols, asking whether the participant considers her/himself in need of further treatment for her/his depression. The item is scored on a scale from 0 (no need for further treatment) to 4 (extensive need for further treatment).

Negative Effects Questionnaire (NEQ-20)

NEQ-20 is a condensed version of the original 32 item self-report questionnaire (46) for monitoring and reporting treatment related adverse and unwanted events. The questionnaire uses a 5-point Likert-scale ranging from "Not at all" to "Extremely" and also includes an open question at the end about other possibly experienced negative or adverse events. The NEQ-20 has exhibited fairness in testing across sociodemographic and shows comparable validity compared to the original 32-items version (47).

Short Revised Children's Anxiety and Depression Scale (RCADS-S)

The RCADS-S (48) is a shortened version of the Spence Child Anxiety Scale, which is a child and caregiver self-report measure of anxiety- and depression-related psychopathology. We will only administer the anxiety subscales, since depression is measured thoroughly by other measures. Subtracting the depression subscale, RCADS-S-C consists of 15 items, reflecting a

single "broad anxiety" dimension. The four-graded scale ranges from 0 = "Never" to 3 = "Always". The 15-item Anxiety Total scale in the shortened version of RCADS, has shown significant correspondence with anxiety diagnostic groups based on structured clinical interviews (48).

Treatment credibility – Adolescent version

Four qualitative questions about treatment credibility will be administered at week 3, asking how well the treatment suits adolescents with depression, how much they believe this treatment will help him/her, if and to what extent they would recommend this treatment to a friend with depression and how much improvement they expect from the treatment. Each item is scored on a 5-point Likert scale, from 1 to 5. The questionnaire takes around 1 minute to complete.

Quick Inventory of Depressive Symptomatology – Adolescent version (QIDS-A_{17})

The QIDS-A_{17} (49) covers the nine DSM-5 symptoms of depression rated in a scale from 0 (none) to 3 (highest) with a sum-range of 0-27 [46]. A total score of 6-10 indicates mild depression, 11-15 moderate, 16-20 severe and 21 and above very severe. Response is defined as a reduction by half of the initial score on QIDS-A_{17}-C. Remission is defined as below 6 points on QIDS-A_{17}-C. The QIDS-A_{17} is a very reliable measure and most discriminating at moderate levels of depression(49).

Work and Social Adjustment Scale – youth version (WSAS-Y)

The WSAS-Y is a 5-item child-rated scale of impaired functioning in school, everyday life, friends and social life, recreation and hobbies and family and close relationships, adapted from the Work and Social Adjustment Scale (50, 51). The scale has excellent internal consistency, test-retest reliability is adequate and has good convergent and divergent validity. Also, the WSAS-Y/P is highly sensitive to change after treatment (51).

5.7.5 SECONDARY CAREGIVER -REPORTED OUTCOMES

Concomitant interventions

To assess what other treatments the adolescent is accessing during the trial period, the caregivers answer a questionnaire at post-treatment and at follow-up assessments with questions about medications (type, dose, indication and time period).

CSQ

The caregiver will answer equivalent questions as the adolescent. See section 5.7.4 for more information.

The Expressed Emotion Adjective Checklist (EEAC)

The EEAC is a validated self-rated questionnaire of the caregiver's positive and negative emotions directed towards the adolescent. The EEAC includes 20 adjectives (each scored 1-8 where 1 indicates never and 8 always).

KIDSCREEN-10 Index
The caregiver will answer the same questions as the adolescent but worded to reflect the adolescent's experience. See section 5.7.4 for more information.

Need for further treatment
The caregiver will answer equivalent questions as the adolescent. See section 5.7.4 for more information.

NEQ-20
The caregiver will answer the same questions as the adolescent but worded to reflect the adolescent's experience. See section 5.7.4 for more information.

RCADS-S
The caregiver will answer the same questions as the adolescent but worded to reflect the adolescent's experience. See section 5.7.4 for more information.

Trimbos Questionnaire for Costs associated with Psychiatric Illness (TiC-P)
The TiC-P (52) will be used to assess healthcare and societal resource use and for cost-of-illness analysis. The questionnaire includes items on healthcare resource use (e.g., healthcare visits), supportive resources (e.g., private tutoring), medications, prescription-free drugs, school absenteeism, academic productivity loss, and parental productivity loss and is frequently used in health economic studies. The TiC-P is a feasible and reliable measure with satisfactory construct validity (52).

Treatment credibility
The caregiver will answer the same questions as the adolescent. See section 5.7.4 for more information.

QIDS-A_{17}-PR
The caregiver will answer the same questions as the adolescent but worded to reflect the adolescent's experience. See section 5.7.4 for more information.

WSAS
The caregiver will answer the same questions as the adolescent but worded to reflect the adolescent's experience. See section 5.7.4 for more information.

6 PROCEDURES

6.1 RECRUITMENT RATE

Recruitment is planned to be conducted within an 18-month period, but will have to be adjusted according to actual recruitment rate. Our sample size calculations show we need to recruit 215 participants. The 18-month period is in practice a 15-month period, if extracting 2.5 months when potential participating families are on holiday (2 weeks around Christmas in 2021- and 8-weeks during summer 2022). During these 15 months, the average recruitment rate needs to be approximately 14 participants per month. As there is often a lag period at the beginning of recruitment, before the advertising campaign takes full effect, we anticipate a slightly slower start to recruitment. To improve inclusion rate, from Autumn 2022 patients will also be recruited directly from BUP Lund.

As there is often a lag period at the beginning of recruitment, before our advertising campaign takes full effect, we anticipate a slightly slower start to recruitment. To assure we are on track with our recruitment rate, we will strive to have recruited a third (approximately 70 patients) after 7 months (approximately 3 participants per week, with holidays excluded), and another 155 patients after 15 months (approximately 6,5 participants per week, with holidays excluded).

Recruitment to our pilot trial (N=32 for 6 months) was slow the first three months when we did not use advertisements (0.5 included per week), but then faster the last three months (2.3 included per week). We will be dependent on frequent advertisements in local media and on using social media in this trial to be able to keep the planned recruitment rate. We will accept referrals from child and adolescent mental health services, primary care and other caregivers, as well as self-referrals from all over Sweden.

6.2 SCREENING AND RECRUITMENT PROCEDURES

1. Adolescents and their caregivers can be self-referred via an online registration form at the project homepage (https://nyheter.ki.se/ar-din-tonaring-deprimerad-ungdomar-13-17-ar-sokes-till-en-studie) or referred to by a clinician.
2. Applicants are then contacted by phone by a psychologist for a broad screening of inclusion and exclusion criteria.
    a. On the recruitment site BUP Lund, recent information from medical records might be used to assist in the screening of the study criteria.
3. In the next step, the adolescent and at least one caregiver will be invited for a more thorough assessment of study eligibility at our unit. If the family do not want to travel to the clinic, or if there is a risk for transmission of the coronavirus, we offer a video assessment.
4. Prior to the diagnostic assessment visit the adolescent and their caregivers are provided with written research participant information and consent form to be

signed on paper or digitally through a secure platform (BASS) by the adolescent and the caregivers. If one of the caregivers is not able to participate at the assessment visit, he/she will be given the opportunity to ask questions about the treatments and participation in the study in prior to the visit.

5. No study specific procedures start before informed consent has been signed by the adolescent and the caregiver/s.
6. At the diagnostic assessment visit, a licensed psychologist will a) verify major depressive disorder diagnosis according to DSM-V, b) assess psychiatric comorbidity, c) decide on inclusion/exclusion and d) assess current severity level of depression and ask about relevant medical history.
    a. On the recruitment site BUP Lund, recent information from medical records might be used to assist in the information gathering prior to the diagnostic assessment, so that patients and their caregivers do not have to answer the same questions repeatedly.
7. If eligible, the adolescent will be offered participation in the study. Patients that fulfil one or more exclusion criteria will be given appropriate healthcare information or referred to other services if needed.
8. Before randomisation, the participants will fill in the baseline measures.
9. Enrolment and randomisation to one of the three interventions (week 0). Randomisation is only allowed if written consent has been filled in by adolescents and both caregivers.

Figure 1. Flow-chart describing the steps from recruitment to enrolment and treatment allocation

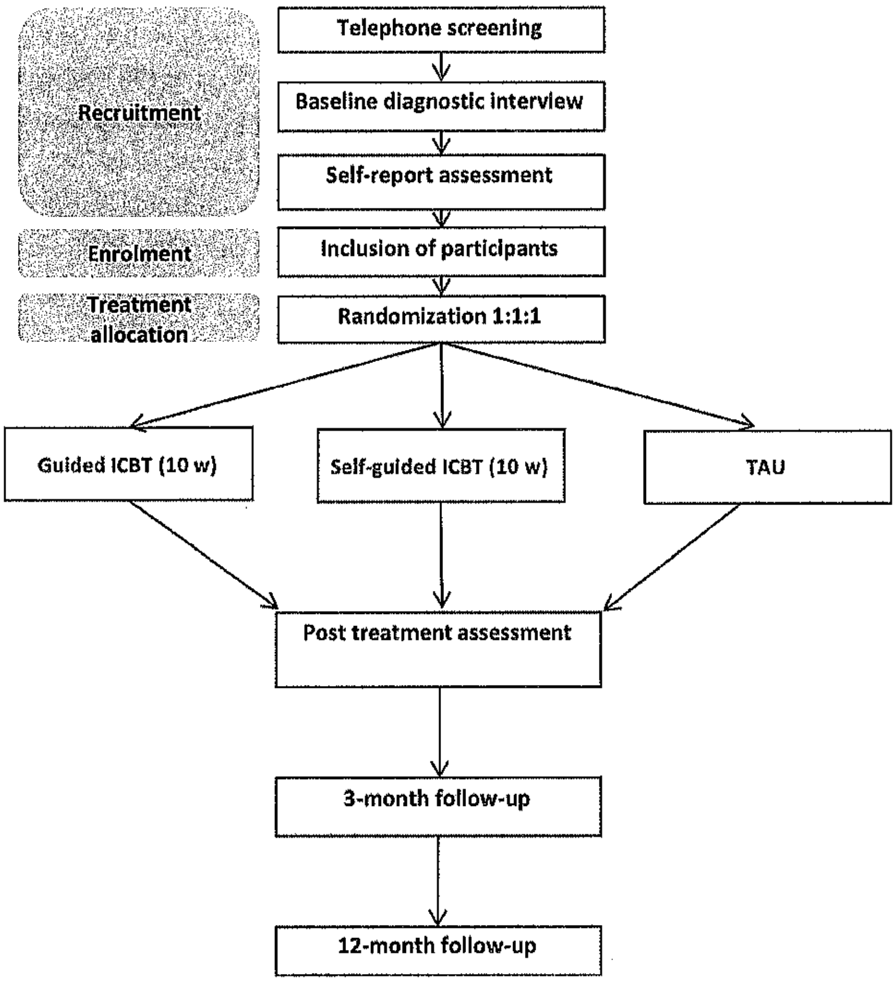

6.3 RANDOMISATION, ENROLMENT AND MASKING

Participants will be randomised at a 1:1:1 ratio to I-BA (guided, self-guided internet-delivered behavioral activation) and a control condition (TAU for depression within CAMHS or pediatric primary care). Randomisation is planned to occur as soon as possible after the diagnostic assessment visit, and never later than 1 month after this visit.

Randomisation will be set up and monitored by the Karolinska Trial Alliance (KTA) using an online randomisation service (ALEA). Randomly varying block sizes of six and nine will be generated using a computer random number generator. We will stratify by two sites, to enable for inclusion of an extra site later on in the trial.

The trial coordinator or co-coordinator will be responsible for enrolment of participants and assigning participants to therapists. Participants will be informed that they will be allocated to one of three arms in the study.

Assessors conducting post- and follow-up assessments will be blind to treatment allocation, until the last follow-up (12FU). The outcome measures are identical for both groups, ensuring that the assessors remain blind. At each follow-up assessment, participants will be reminded by their assessor to not reveal their arm allocation. To measure blinding integrity, all assessors will record whether the participating families inadvertently reveal their group allocation, and subsequently guess each participant's treatment allocation at each assessment point and motivate their choice. The blinding will be broken after the trial's final participant has finished his/her 3-month follow-up assessment (primary endpoint). Blinding of individual participants will be kept for blind assessors until the 12-mont follow-up assessment. The trial will end when the trial's final participant has finished his/her 12-month follow-up assessment.

In case of a medical emergency, we will refer participants to appropriate health care. The trial coordinator will be in charge of following up the incident. This whole procedure can be done without unblinding the outcome assessors, so an emergency unblinding system is not required for the trial.

6.4 TREATMENT

6.4.1 GUIDED AND SELF-GUIDED I-BA

The intervention arms consist of internet-delivered BA with (guided) and without therapist-support (self-guided) and involve both the adolescent and their caregiver. The adolescent and the caregiver are provided with their own separate programs and login to the platform.

Each of the I-BA interventions consists of 8 modules, delivered over a maximum of 10 weeks. In certain circumstances (e.g., illness or holidays), participants in guided ICBT can pause their therapist-support for one or two weeks. After 10 weeks, all modules will be made available for the participant (regardless of how many modules s/he has completed). The adolescent and caregiver can continue to access all treatment modules for the whole follow-up period (3 months), but without therapist-support.

During guided I-BA, participants have regular contact with a trained therapist inside the platform. The therapist provides feedback, answer questions, and reminds you to complete the next module if required. Communication is possible via traditional text messages (resembling e-mail) or via comments on specific worksheets. When the participant receives a message on the platform, s/he also gets an SMS reminder to her/his phone. The therapist usually logs in daily (on workdays; at minimum every 48 hours). Therapist time is automatically logged in to the platform. Participants are typically in contact with their therapist several times a week. Phone calls are possible if the participant/therapist for a certain reason feels it is needed but are generally kept to a minimum. The participants are welcome to contact their therapist at any time, who will reply during office hours.

An overview of the treatment is presented in **Table 1**. The treatment manual, developed by Grudin, consists of evidence-based interventions adapted to an online format inspired by previously published treatment manuals on behavioral activation for adolescents (36, 53). The main goal of the BA is to increase engagement in values-based activities and decrease avoidant behaviors that serve to maintain depression. The adolescent will learn about depression, how behavioral activations works, monitor their own activities, plan and do more values-based activities and deal with obstacles to getting in touch with positive reinforcement, such as avoidance behaviors.

The adolescents will also learn about common sleep problems in depression and how they can improve their sleep. Sleep disturbances commonly co-occur with depression in adolescents (54). However, research on this area is lagging behind and the Board of Health and Welfare provides no guidelines for treatment of insomnia when co-occurring with depression (55). The British National Institute for Health and Care Excellence's (NICE) guidelines for treatment of depression states that patients with sleep problems in depression should be offered advice on sleep hygiene (56). Therefore, we decided to include brief psychoeducation on sleep problems and advice on adaptive sleep behaviors (e.g., keeping regular sleep hours) early in treatment, to enable continuous work with establishing better sleep routines during the rest of the treatment.

The self-guided I-BA is identical to the guided I-BA intervention, however without therapist support. To ensure patient-safety, there will be clear instructions to the patients and primary caregivers how to get in contact with the study team in case of acute problems, and there will be clinical routines to detect and manage deterioration or suicidal tendencies.

We included a caregiver program in I-BA because caregivers are a significant part of the adolescents' daily life and can provide support with treatment assignments. Caregivers may also be part of an ineffective communication pattern that contributes to maintained depression. Involving caregivers facilitates modification of these unhelpful patterns. Caregivers are also commonly engaged in other BA-protocols for adolescents (53, 57). The caregiver program is consistent with principles from Parent Management Training (PMT; (58, 59), that teach caregivers positive parenting skills, aiming at strengthening the relationship between caregivers and their children.

The caregiver program consists of 8 modules, including psychoeducation about depression, how to support their adolescent suffering from depression, reviews of the content in the adolescent program, caregiver coping strategies, collaborative problem-solving and effective communication skills. Both the adolescent and the caregiver in guided I-BA have individual access to the same therapist. See **Table 1** below for more details on the treatment content.

Table 1. Treatment overview of I-BA

Module	Adolescent	Caregiver
1	Introduction to I-BA. Psychoeducation on depression. Rationale for BA. *Homework*: **activity monitoring.**	Introduction to I-BA. Psychoeducation on depression. Rationale for BA. Learn about **common parental traps.** *Homework*: notice one's parental behaviors when the adolescent shows depressive behaviors. Discuss with the adolescent how to collaborate in treatment.
2	Values assessment. Set treatment goals. *Homework*: **activity scheduling.**	**Facilitate and encourage values-based activation;** communication skills I: **validate your child's feelings.** *Homework:* practice validating others' and your child's emotions, encourage values-based activation.
3	Continued **values-based activation. Psychoeducation on sleep.** *Homework*: activity scheduling and sleep hygiene.	**Spending positive time** with your adolescent. *Homework*: suggest positive time with your adolescent.
4	Continued **values-based activation.** Identify and overcome barriers to activation through **identifying and overcoming avoidance.** *Homework*: activity scheduling, sleep hygiene and practice overcoming avoidance.	Communication skills II: How to **avoid and manage conflicts.** *Homework*: practice conflict management.
5	Continued **values-based activation.** Overcome barriers to activation through **shifting focus to the present situation.** *Homework*: activity scheduling, sleep hygiene and practice shifting focus.	**Take care of yourself** as a caregiver supporting a child with depression. *Homework*: take care of yourself.
6	Continued **values-based activation. Problem solving.** *Homework*: activity scheduling, sleep hygiene and problem-solving practice.	**Collaborative problem-solving.** *Homework*: practice collaborative problem-solving
7	**Putting it all together.** *Homework*: activity scheduling.	**Putting it all together.** *Homework*: choose two tasks from previously introduced skills.
8	Treatment summary. **Relapse prevention.** Make an evaluation of the treatment.	Course summary. **Relapse prevention.** Make an evaluation of treatment.

6.4.2 TREATMENT AS USUAL (TAU)

Our control condition represents treatment as usual. Participants randomised to TAU, will be referred to the local CAMHS or primary care unit for children and youths and will be free to receive any treatment, either psychosocial, medical or the combination of both. The

content of TAU and the treatment techniques used, will be collected by clinician-interview with the caregiver at 3-months follow-up.

6.4.3 THERAPIST TRAINING, SUPERVISION AND MONITORING

All therapists will receive training before the trial starts. They will be introduced to the use of the platform and the I-BA protocol during a one-day training by the treatment constructor (Grudin). Regular supervision will be held to discuss treatment progress for individual patients and follow up on clinical needs and potential adverse events. Protocol violations will be recorded.

6.5 SUBSEQUENT ASSESSMENTS

6.5.1 ASSESSMENTS DURING TREATMENT

Data is collected weekly for QIDS-A_{17} to monitor depressive symptoms and suicidality, and BADS-SF, to evaluate changes in the presumed therapeutic processes. See **Table 2** for information regarding which measures are administered at each time point. For these assessments, there is a 7-day time period for data collection.

6.5.2 POST-TREATMENT ASSESSMENT

The post-treatment assessment is conducted face-to-face at the clinic, or video if the family cannot travel to the clinic or prefer so. Adolescent and caregiver-reported questionnaires are answered online directly to our trial database BASS. See **Table 2** for information regarding which measures are administered at this time point. The post-treatment assessment is planned to occur as quickly as possible after the end of the 10-week treatment, and never later than 2 months after the treatment.

6.5.3 FOLLOW-UP ASSESSMENTS

Follow-ups will be administered 3 and 12 months after the end of the 10-week I-BA treatment period, or 11 weeks from when the referral is sent for TAU. As with the post-treatment assessment, these assessments are conducted face-to-face at the clinic or over video. The CDRS-R interview will be audio-recorded. Adolescent- and caregiver-reported questionnaires are answered online directly to our trial database BASS, preferably prior to the face-to-face assessment. See **Table 2** for information regarding which measures are administered at each time point. For these follow-ups, there is a -1 month/+2-month time period for data collection. After completing the three-month follow-up, participants are given a gift card worth 400 SEK.

Table 2. Assessments points for each measure

Assessment points / Outcomes	Baseline	Weekly during treatment	Post-treatment	3-month follow-up **(primary endpoint)**	12-month follow-up
**Baseline measures**					
Demographic data (clinician)	X				
Demographic data (caregiver)	X				
MINI-KID	X				
DSHI	X				
**Clinician-rated**					
BASS platform usage data + phone time			X		
Blindness checks			X	X	X
CDRS-R	X		X	X	X
CGI-S	X		X	X	X
CGI-I			X	X	X
CGAS	X		X	X	X
iiPAS			X		
**Child-reported**					
QIDS-A_{17}	X	X	X	X	X
ARI	X		X	X	X
ASA	X		X	X	X
BADS	X	X	X	X	X
Concomitant interventions			X	X	X
CSQ			X		
ISI	X		X	X	X
KIDSCREEN-10	X		X	X	X
Need for further treatment				X	
NEQ			X	X	
RCADS-S	X		X	X	X
Treatment credibility		X (week 3)			
WSAS	X		X	X	X
**Caregiver-reported**					
QIDS-A_{17}	X	After 5 weeks	X	X	X
Concomitant interventions			X	X	X
CSQ			X		
EEAC	X		X	X	X
KIDSCREEN	X		X	X	X
Need for further treatment				X	
NEQ			X	X	
RCADS-S	X		X	X	X
TIC-P	X		X	X	X
Treatment credibility		After 3 weeks			
WSAS	X		X	X	X

Note: For full names of measures, see section 5.7.

6.6 END OF TRIAL

The trial will end when the final data (at the 12-month follow-up) has been collected for the final participant.

6.6.1 PARTICIPANT WITHDRAWAL FROM TRIAL

Participants are free to withdraw from the trial at any point. After the withdrawal, participants will not be requested to complete any further measures but will be asked to provide non-obligatory feedback regarding their reason for withdrawal. This reason (if given) will be logged for reporting purposes. Once participants have withdrawn from the trial, it will not be possible to re-enter or resume treatment. Withdrawn participants will not be replaced in the trial.

6.6.2 DISCONTINUATION OF TRIAL

Outcome measures will not be analyzed until all data from the primary endpoint is collected and will therefore not inform decisions to stop the research. However, serious adverse events (see section 9.1 for definitions) will be reviewed, and if there is any indication that these are linked to the intervention consideration will be given to discontinuing the trial on the advice of the research group and trial Sponsor. Failure to recruit could also be a reason to stop the trial.

6.6.3 CONCOMITANT INTERVENTIONS

Any medication for the indication of depression (primarily antidepressants), ADHD (primarily central stimulants), and neuroleptics is required to have been stable for the last six weeks prior to inclusion in the trial. Additionally, participants in the I-BA groups are encouraged (if possible, according to her/his treating clinician) not to alter her/his medication until after the 3-month follow-up. Participants must not have received previous evidence-based psychological treatment (CBT or IPT) or BA for depression within 12 months prior to inclusion into the trial. Further, the participants in the ICBT groups are asked to not start any parallel psychological treatment until after the 3-month follow-up. Data on the use of medication and psychological interventions are collected at each assessment point. Any deviations to protocol will be noted and potentially considered in the statistical analysis.

6.7. QUALITY CONTROL

The trial will be conducted according to good clinical practice principles (GCP). Data quality and safety aspects will be regularly monitored by an independent party, the Karolinska Trial Alliance (KTA). Statistical analyses will be conducted under the guidance of the Karolinska Institutet Biostatistics Core Facility (www.biostatcore.ki.se).

7 DATA MANAGEMENT

All aspects of data management of the trial will comply with the General Data Protection Regulation (GDPR) and good clinical practice (GCP). Notes will be made in the medical record software Take Care (included participants only) according to BUP Stockholm standard routines for research trials on internet-delivered treatment.

7.1 DATA COLLECTION AND HANDLING

Data will be collected both manually (paper Case Report Forms (CRFs)) and digitally (adolescent- and caregiver-reported questionnaires completed via the internet directly into the trial database BASS). Some data will also be extracted digitally from the treatment platform, and then entered to BASS.

The CRFs will not bear the participant's name, instead the trial identification number will be used for identification. CRFs will be stored securely in a locked file cabinet. Data from the CRFs will consecutively be entered manually into BASS by a member of the research team. The delegation log will identify all those personnel with responsibilities for data collection and handling, including those who have access to the trial database. Audio- and video recordings of assessments will be stored on an external hard drive in a locked file cabinet. The recordings will be used for methodology checks, re-ratings if blinding is broken and future training of trial assessors.

7.1.1 BASS

BASS is the name of the treatment platform and trial online database. BASS is used both for manual entry of clinician assessed/gathered information, as well as for digital administration of written consent as well as adolescent- and caregiver-reported questionnaires. Regardless of the method above, all data goes into the same database. BASS has a fine-grained privilege-system which ensures that personnel only can view information and edit settings that pertain to their role in the research project, including only viewing their relevant participants. All data sent between the platforms and the user are encrypted using a 2048-bit SSL certificate. The system is setup so that only a port 80 and 443 is visible on the internet to the public.

The BASS system store names of participants and telephone numbers (for the use of sending SMS). Logging into the BASS platform requires username, password, and a temporary code sent by SMS (i.e., two-factor authentication which is required for systems storing sensitive data). Once the trial is finished, names and telephone numbers will be removed from BASS.

When the trial has ended, data will be extracted from BASS by the trial coordinator (Grudin), then properly prepared (by a member of the research team, including adding a variable for the treatment allocation), and then securely transferred to the parts responsible for statistical analyses (see section 8). Some analyses will also be made locally by the research team.

7.1.2 TEAMS

Teams is the software solution that will be used for the videoconference assessments. Teams can be used in the common modern browsers (e.g., Chrome, Safari and Firefox), as well as an external application. Through Teams, an e-mail is sent from the research team with a link from where the participant initiates the video conference. Teams offers password-protected meetings, a lobby function and end-to-end cryption.

8 STATISTICAL ANALYSIS

Analysis of trial feasibility: Descriptive statistics on trial feasibility data, e.g., number of excluded patients and reasons to exclude, participant refusals and causes for refusal (e.g., did not want to be randomised to TAU), withdrawal, and attrition rates, and number of patients who declined to participate and reasons why.

Summary of baseline data: All baseline variables will be summarized by randomised group.

Analysis of acceptability of guided and self-guided I-BA: Descriptive statistics on treatment satisfaction, treatment credibility and therapist time.

Analysis of clinical efficacy: We will follow the CONSORT guidelines in reporting and analyzing our data. The quantitative analyses of continuous measures (primary and secondary outcomes) are conducted according to the "intent-to-treat" principle, including all randomised participants in hierarchical linear mixed-models analysis. Linear mixed models are also an adequate method to handle missing data. Cost data will include resource use costs (e.g., hospital visits, medication and absence from school, caregiver's absence from work) and cost of I-BA or TAU. Cost differences between guided I-BA, self-guided I-BA and TAU can be calculated, as well as the incremental cost effectiveness ratio (ICER). The ICER is a global estimate of cost-effectiveness and represents the additional cost that is associated with the additional clinical effect in one intervention compared with another.

9 RECORDING AND REPORTING OF ADVERSE EVENTS AND REACTIONS

9.1 DEFINITIONS

Table 3. Definitions of adverse and serious adverse events relevant for this trial (60)

Term	Definition
Adverse Events (AE)	Side effects that are harmful. Not necessarily a causal relationship with the treatment. For example:<br>- a psychological event (e.g., depressed mood).<br>- an increase in the severity or frequency of a pre-existing symptom or condition (e.g., increased self-injury)
Serious Adverse Event (SAE)	Any untoward medical occurrence that:<br>- results in death (e.g., suicide)<br>- is life-threatening (e.g., suicide attempt)<br>- requires hospitalization or prolongation of existing hospitalization (e.g., due to high suicide risk)<br>- results in persistent or significant disability or incapacity<br>- is otherwise considered medically significant by the investigator

9.2 EXPECTED ADVERSE EVENTS

The following negative treatment-related events are considered expected in the proposed trial given previous research on ICBT for depression (61) and other disorders (62):

- Increased depressive symptoms
- Thoughts of death and suicide
- Increased stress due to tempo and workload
- Feeling worse while processing treatment content
- Increased irritability
- Increased anxiety/stress
- Increased tiredness/fatigue
- Increased/decreased sleep
- Headaches
- Not having confidence in the treatment
- Not feeling that the treatment produces any results
- Feeling that the treatment is not always motivating
- Not always understanding the treatment

It is important to note that adverse events may also be symptoms of the underlying condition, rather than the intervention itself. Adverse events will be closely monitored by the therapist and reported in accordance to the procedures outlined in this protocol.

9.3 ASSESSMENT OF ADVERSE EVENTS

A. ASSESSMENT OF CAUSAL RELATIONSHIP

The assessment of the relationship between adverse events and the administration of the treatment is a decision based on all available information. The final decision is taken by the PI, or if she is not present team member and medical doctor Anna Ohlis. If the event is a result of the administration of any of the research procedures, then it will be classed as related.

All AE will be categorized either as likely related, possibly related, or not related, in accordance with the definitions below:

Likely related: Clinical event occurring within a reasonable time after administration of the intervention. It is unlikely that the event can be attributed to underlying disease or medications, but is most likely caused by the intervention.
Possibly related: Clinical event occurring within a reasonable time after administration of the intervention. The event could be explained by the intervention, but there is insufficient information to determine the relationship. The event could be explained by an underlying disease or medications.
Not related: Clinical event that is not reasonably related to the intervention. The event is unlikely related to the intervention and can be explained by medications or underlying disease.

B. ASSESSMENT OF INTENSITY

In addition to assessing the causal relationship between administration of the intervention and AE, an assessment of the intensity of the event is required. The following classifications will be used:
Mild: The adverse event is mild and transient in nature and does not affect the subject's normal life, and does not require any intervention or treatment (e.g., temporary insomnia, mild headache).
Moderate: The adverse event causes deterioration of function but does not affect health. The event can be sufficiently unpleasant and interferes with normal activities but does not completely obstruct them. May require interventional treatment (e.g., fever requiring antipyretic medication).
Severe – event results in significant symptoms that prevents normal daily activities; may require hospitalization or invasive intervention (e.g., anemia resulting in blood transfusion).

C. ASSESSMENT OF SERIOUSNESS

The investigator is responsible for assessing the seriousness (**serious** or **non-serious**). If the incident is considered serious, this should be reported as a serious adverse event (SAE) by the investigator to the sponsor.

9.4 HANDLING OF ADVERSE EVENTS

Adverse events will be noted by the trial coordinator in a specific log (including date, recorded clinical symptoms, and a brief description of the event). The research team could be notified about adverse events in numerous ways, including through direct (text or telephone) communication with the participant, or at follow-up assessments. Regular meetings (treatment conferences) will be held within the research team where treatment progress and potential adverse events are discussed. Cut-off scores on the suicidal item in QIDS-A_{17} (self-rated depressive symptoms), might also be indicators of adverse events, and thus be registered in the AE-log. Scores of 2 or 3 points on the suicide item corresponding to a yes to "I think of suicide or death several times a week for several minutes" or "I think of suicide or death several times a day in some detail, or I have made specific plans for suicide or have actually tried to take my life", will automatically raise a flag in the BASS system, to directly notify members of the research team to follow this up (via the telephone) with the participant.

SAEs will be recorded in the patient's medical record (Take Care), as well as in the trial coordinator's log. Appropriate action will be taken in the case of SAE, making sure the participant will get in contact with suitable health care services. Events will be considered as potentially treatment-related up to the 3-month follow-up, where the reporting of adverse events will terminate. The PI will immediately notify the Sponsor of any events of SAE. The PI and sponsor will work together to identify the extent of the SAE and to determine what urgent safety measures are required.

10 PUBLIC AND PATIENT INVOLVEMENT

Users are involved in the further treatment development before this study. Involving young people with experience of depression is vital to develop an intervention that young people will want to use, and to design an evaluation inclusive of the perspectives of patients and caregivers. During the developmental phase of the treatment and before the pilot study we involved patient representatives to give feedback on the treatment content as well as on information about the study. We are keen to use a language that is inclusive (e.g., LGBTQ) and understandable, animations that are useful and to use examples that are representative of an adolescent view of the world and him/herself. After completion of the pilot study, we also conducted qualitative interviews to investigate patient experiences of I-BA for depression. Specific changes were made to the treatment based on received patient feedback.

11 DATA SHARING

We will potentially share trial data with other depression researchers around the world. The use will primarily be for similar objectives as in the current trial, for instance to combine treatment outcome data for meta-analyses. Shared data will be de-identified. The possibility of future data sharing is mentioned in the informed consent form.

12 ETHICAL CONSIDERATIONS

ICBT differs from face-to-face therapy only in the format and mode of delivery, but still contains the same evidence-based ingredients and principles as recommended in guidelines for depression. ICBT for depression has good empirical support for adults, and our assumption is that it is likely useful and effective for adolescents as well.

Verbal information about the study and written informed consent forms will be provided to the adolescent and both caregivers prior to baseline assessment. All participant families will be volunteers, competent to provide informed consent. Participants may withdraw from the trial at any time.

Depression is a high-risk group to work with and patient safety will be a high priority in this study. Participants in all treatment arms will answer brief measures of depression weekly during the intervention, allowing depressive symptoms and suicidal ideation to be monitored. A thorough diagnostic and mental health status examination before enrolment of participants will prevent patients with more immediate needs or risks from being included in the study and offered alternative treatment.

The treatment target of the I-BA-interventions is to decrease depressive symptoms and increase daily functioning through behavior change. Therefore, we expect commonly experienced negative treatment effects for some participants, such as occasional discomfort due to approaching previously avoided and difficult situations, occasional stress due to tempo and workload in treatment, and at times feeling worse while processing treatment content. The treatment model encourages the patients to approach situations that may evoke temporary discomfort, e.g., reconnect with a friend that you have not spoken to in a while. A thorough rationale will be given for each component of the treatment as to prepare the participant for the expected temporary discomfort and other prerequisites of the treatment. Behavior change will also happen in small steps, in accordance with the values and goals of the adolescent.

This study will be conducted within regular health care, which means that the project will use medical records and be able to quickly refer adolescents in need of more help. We will also have weekly conferences discussing patients and a child- and adolescent psychiatrist will be present at these conferences.

Adverse events will be carefully monitored in all three groups throughout the trial. Where necessary, the participant's local teams or GP will be informed about the event. The TAU arm means contact with regular health care which will ensure patient safety.

Participants may worry about computer safety and confidentiality. To prevent this, families will receive information about the risks and precautions that are being taken when using communication technology (e.g., encrypted server technology and double authentication with password and SMS-code).

Each participant will be assigned a unique trial identification number at the point of randomisation. This number will be recorded on all paper datasheets and in the electronic trial database. A hard copy of a record sheet linking patient identity, contact details, screening number, and trial identification number for all participants will be kept securely in a locked filing cabinet separate from datasheets. This will also be stored in a password protected electronic database, which only will be accessible to authorized users. All data will be kept secure at all times and maintained in accordance with the requirements of GCP regulations. ELN will be used to log all significant decisions during the trial.

In conclusion, the risks for the participants are considered to be limited and temporary. The risks will be far outweighed by the potential benefits for the participant and the scientific value of the study.

13 IMPLICATIONS

The number of adolescents seeking professional help for mental health problems is increasing and, despite advances in treatment, large unmet treatment needs remain. The healthcare system is under pressure due to rising healthcare costs. Moreover, quality and access to healthcare are inequitably distributed across the country. This project addresses these challenges. The project could add a potentially cost-effective way to deliver evidence-based psychological treatments for depression and could increase the availability of such treatments by breaking down geographical barrier. ICBT could decrease societal and health care costs through more efficient administration, reduced waiting times, reduced therapist resources, and less absence from school and work for the families. If ICBT could be unguided, even more adolescents with depression could receive help. In addition, ICBT is an inclusive and flexible treatment that emphasizes patient autonomy and allows them to decide when and where to work with the treatment. The COVID-19 pandemic has further highlighted the need to offer cost-effective interventions that can be delivered remotely. If the I-BA treatment for depression proves to be effective and cost-effective, we aim to implement it nationally via the Stöd och behandling platform (SOB) and the new Internet CBT unit for youths, which is steered by co-applicant Vigerland.

14 FINANCE

The trial is financed by Kavli (registration number 4-3124/2018) and Frimurare Barnhuset in Stockholm.

15 PUBLICATION PLANS

We plan to publish two primary papers. Paper 1 will report on efficacy and cost-effectiveness at the primary endpoint (3 months post-treatment). Paper 2 will report long term follow up (12 months post-treatment). These papers will be submitted to general or specialty medical and psychological science journals.

16 REFERENCES

1. American Psychiatric A. Diagnostic and statistical manual of mental disorders : DSM-5. Fifth edition. ed: Arlington, VA : American Psychiatric Publishing; 2013.
2. Ferrari AJ, Charlson FJ, Norman RE, Patten SB, Freedman G, Murray CJ, et al. Burden of depressive disorders by country, sex, age, and year: findings from the global burden of disease study 2010. PLoS medicine. 2013;10(11):e1001547.
3. Mathers C, Fat DM, Boerma JT, World Health O. The global burden of disease 2004 update. Geneva, Switzerland: Geneva, Switzerland : World Health Organization; 2008.
4. Costello EJ, Egger H, Angold A. 10-year research update review: the epidemiology of child and adolescent psychiatric disorders: I. Methods and public health burden. J Am Acad Child Adolesc Psychiatry. 2005;44(10):972-86.
5. Costello EJ, Mustillo S, Erkanli A, Keeler G, Angold A. Prevalence and Development of Psychiatric Disorders in Childhood and Adolescence. Archives of general psychiatry. 2003;60(8):837-44.
6. Jonsson U, Bohman H, Hjern A, Von Knorring L, Olsson G, Von Knorring AL. Subsequent higher education after adolescent depression: A 15-year follow-up register study. Eur Psychiatry. 2010;25(7):396-401.
7. Jonsson U, Bohman H, Von Knorring L, Olsson G, Paaren A, Von Knorring AL. Mental health outcome of long-term and episodic adolescent depression: 15-year follow-up of a community sample. Journal of Affective Disorders. 2011;130(3):395-404.
8. Kessler RC. The Costs of Depression. Psychiatr Clin North Am. 2012;35(1):1-14.
9. Neufeld SAS, Dunn VJ, Jones PB, Croudace TJ, Goodyer IM. Reduction in adolescent depression after contact with mental health services: a longitudinal cohort study in the UK. The Lancet Psychiatry. 2017;4(2):120-7.
10. National guidelines for depression and anxiety disorder care: support for management and management (Nationella riktlinjer för vård vid depression och ångestsyndrom: stöd för styrning och ledning). . Stockholm: The National Board of Health and Welfare., Socialstyrelsen; 2017.
11. Cuijpers P, Van Straten A, Andersson G, Van Oppen P. Psychotherapy for Depression in Adults: A Meta-Analysis of Comparative Outcome Studies. J Consult Clin Psychol. 2008;76(6):909-22.
12. David E, Lisa W, Annemieke Van S, Pim C, David R, Simon G. Behavioural activation for depression

an update of meta-analysis of effectiveness and sub group analysis. PLoS One. 2014;9(6):e100100.
13. Jacobson NS, et al. A Component Analysis of Cognitive-Behavioral Treatment for Depression. J Consult Clin Psychol. 1996;64(2):295-304.
14. Dimidjian S, Hollon SD, Dobson KS, Schmaling KB, Kohlenberg RJ, Addis ME, et al. Randomized Trial of Behavioral Activation, Cognitive Therapy, and Antidepressant Medication in the Acute Treatment of Adults with Major Depression. J Consult Clin Psychol. 2006;74(4):658-70.

15. de Haan AM, Boon AE, de Jong JTVM, Hoeve M, Vermeiren RRJM. A meta-analytic review on treatment dropout in child and adolescent outpatient mental health care. Clinical Psychology Review. 2013;33(5):698-711.
16. Collins KA, Westra HA, Dozois DJA, Burns DD. Gaps in accessing treatment for anxiety and depression: Challenges for the delivery of care. Clinical Psychology Review. 2004;24(5):583-616.
17. Essau CA. Frequency and patterns of mental health services utilization among adolescents with anxiety and depressive disorders. Depress Anxiety. 2005;22(3):130-7.
18. Andersson E, Enander J, Andrén P, Hedman E, Ljótsson B, Hursti T, et al. Internet-based cognitive behaviour therapy for obsessive–compulsive disorder: a randomized controlled trial. Psychol Med. 2012;42(10):2193-203.
19. Hedman E, Ljotsson B, Lindefors N. Cognitive behavior therapy via the Internet: a systematic review of applications, clinical efficacy and cost-effectiveness. Expert Rev Pharmacoecon Outcomes Res. 2012;12(6):745-64.
20. Andersson G, Cuijpers P, Carlbring P, Riper H, Hedman E. Guided Internet-based vs. face-to-face cognitive behavior therapy for psychiatric and somatic disorders: a systematic review and meta-analysis. World Psychiatry. 2014;13:288–95.
21. Sethi S, Campbell AJ, Ellis LA. The Use of Computerized Self-Help Packages to Treat Adolescent Depression and Anxiety. Journal of Technology in Human Services. 2010;28(3):144-60.
22. Makarushka MM. Efficacy of an Internet-based intervention targeted to adolescents with subthreshold depression: University of Oregon; 2011.
23. Topooco N, Berg M, Johansson S, Liljethorn L, Radvogin E, Vlaescu G, et al. Chat- and internet-based cognitive-behavioural therapy in treatment of adolescent depression: randomised controlled trial. Bjpsych Open. 2018;4(4):199-207.
24. Topooco N, Bylehn S, Nysater ED, Holmlund J, Lindegaard J, Johansson S, et al. Evaluating the Efficacy of Internet-Delivered Cognitive Behavioral Therapy Blended With Synchronous Chat Sessions to Treat Adolescent Depression: Randomized Controlled Trial. J Med Internet Res. 2019;21(11).
25. Calear AL, Christensen H. Systematic Review of School-Based Prevention and Early Intervention Programs for Depression. J Adolesc. 2010;33(3):429-38.
26. Wright B, Tindall L, Littlewood E, Allgar V, Abeles P, Trépel D, et al.
27. Poznanski E, Mokros, H. Children's depression rating scale-revised (CDRS-R). Los Angeles, CA: Western Psychological Services; 2001.
28. Gold SM, Enck P, Hasselmann H, Friede T, Hegerl U, Mohr DC, et al. Control conditions for randomised trials of behavioural interventions in psychiatry: a decision framework. The Lancet Psychiatry. 2017;4(9):725-32.
29. Sheehan DV, Sheehan KH, Shytle RD, Janavs J, Bannon Y, Rogers JE, et al. Reliability and Validity of the Mini International Neuropsychiatric Interview for Children and Adolescents (MINI-KID). The Journal of Clinical Psychiatry. 2010;71(03):313-26.
30. Gratz K. Measurement of Deliberate Self-Harm: Preliminary Data on the Deliberate Self-Harm Inventory. Journal of Psychopathology and Behavioral Assessment. 2001;23(4):253-63.
31. Poznanski EO, Cook SC, Carroll BJ. A depression rating scale for children. Pediatrics. 1979;64(4):442.
32. Mayes TL, Bernstein IH, Haley CL, Kennard BD, Emslie GJ. Psychometric Properties of the Children's Depression Rating Scale–Revised in Adolescents. J Child Adolesc Psychopharmacol. 2010;20(6):513-6.
33. Jeon S, Walkup JT, Woods DW, Peterson A, Piacentini J, Wilhelm S, et al. Detecting a clinically meaningful change in tic severity in Tourette syndrome: a comparison of three methods. Contemp Clin Trials. 2013;36(2):414-20.
34. Guy W. ECDEU assessment manual for psychopharmacology: US Department of Health, and Welfare; 1976.
35. Busner J, Targum SD. The clinical global impressions scale: applying a research tool in clinical practice. Psychiatry (Edgmont (Pa : Township)). 2007;4(7):28.

36. McCauley E, Gudmundsen G, Schloredt K, Martell C, Rhew I, Hubley S, et al. The Adolescent Behavioral Activation Program: Adapting Behavioral Activation as a Treatment for Depression in Adolescence. J Clin Child Adolesc Psychol. 2016;45(3):291-304.
37. Shaffer D, Gould MS, Brasic J, Ambrosini P, Fisher P, Bird H, et al. A Children's Global Assessment Scale (CGAS). Archives of General Psychiatry. 1983;40(11):1228-31.
38. Green B, Shirk S, Hanze D, Wanstrath J. The Children's Global Assessment Scale in clinical practice: an empirical evaluation. Journal of the American Academy of Child and Adolescent Psychiatry. 1994;33(8):1158.
39. Lenhard F, Mitsell K, Jolstedt M, Vigerland S, Wahlund T, Nord M, et al. The Internet Intervention Patient Adherence Scale for Guided Internet-Delivered Behavioral Interventions: Development and Psychometric Evaluation. J Med Internet Res. 2019;21(10):e13602-e.
40. Watson R, McCabe C, Harvey K, Reynolds S. Development and validation of a new adolescent self-report scale to measure loss of interest and pleasure: The Anhedonia Scale for Adolescents. Psychological assessment. 2021;33(3):201-17.
41. Stringaris A, Goodman R, Ferdinando S, Razdan V, Muhrer E, Leibenluft E, et al. The Affective Reactivity Index: A Concise Irritability Scale for Clinical and Research Settings. Journal of Child Psychology and Psychiatry. 2012;53(11):1109-17.
42. Manos RC, Kanter JW, Luo W. The Behavioral Activation for Depression Scale-Short Form: Development and Validation. Behav Ther. 2011;42(4):726-39.
43. Larsen DL, Attkisson CC, Hargreaves WA, Nguyen TD. Assessment of client/patient satisfaction: Development of a general scale. Eval Program Plann. 1979;2(3):197-207.
44. Bastien CH, Vallières A, Morin CM. Validation of the Insomnia Severity Index as an outcome measure for insomnia research. Sleep Med. 2001;2(4):297-307.
45. Ravens-Sieberer U, Erhart M, Rajmil L, Herdman M, Auquier P, Bruil J, et al. Reliability, construct and criterion validity of the KIDSCREEN-10 score: a short measure for children and adolescents' well-being and health-related quality of life. Qual Life Res. 2010;19(10):1487-500.
46. Alexander R, Anders K, Johanna B, Gerhard A, Per C. Negative Effects of Psychological Treatments: An Exploratory Factor Analysis of the Negative Effects Questionnaire for Monitoring and Reporting Adverse and Unwanted Events. PLoS One. 2016;11(6):e0157503.
47. Rozental A, Kottorp A, Forsström D, Månsson K, Boettcher J, Andersson G, et al. The Negative Effects Questionnaire: psychometric properties of an instrument for assessing negative effects in psychological treatments. Behav Cogn Psychother. 2019;47(5):559-72.
48. Ebesutani C, Reise SP, Chorpita BF, Ale C, Regan J, Young J, et al. The Revised Child Anxiety and Depression Scale-Short Version: scale reduction via exploratory bifactor modeling of the broad anxiety factor. Psychol Assess. 2012;24(4):833-45.
49. Bernstein IH, Rush AJ, Trivedi MH, Hughes CW, Macleod L, Witte BP, et al. Psychometric properties of the Quick Inventory of Depressive Symptomatology in adolescents. Int J Methods Psychiatr Res. 2010;19(4):185-94.
50. Mundt JC, Marks IM, Shear MK, Greist JH. The Work and Social Adjustment Scale: a simple measure of impairment in functioning. The British journal of psychiatry : the journal of mental science. 2002;180:461.
51. Jassi A, Lenhard F, Krebs G, Gumpert M, Jolstedt M, Andrén P, et al. The Work and Social Adjustment Scale, Youth and Parent Versions: Psychometric Evaluation of a Brief Measure of Functional Impairment in Young People. Child Psychiatry Hum Dev. 2020;51(3):453-60.
52. Bouwmans C, De Jong K, Timman R, Zijlstra-Vlasveld M, Van der Feltz-Cornelis C, Tan SS, et al. Feasibility, reliability and validity of a questionnaire on healthcare consumption and productivity loss in patients with a psychiatric disorder (TiC-P).(Report). BMC Health Serv Res. 2013;13(1).
53. Pass L, Lejuez CW, Reynolds S. Brief Behavioural Activation (Brief BA) for Adolescent Depression: A Pilot Study. Behav Cogn Psychother. 2018;46(2):182-94.

54. Sivertsen B, Harvey A, Lundervold A, Hysing M. Sleep problems and depression in adolescence: results from a large population-based study of Norwegian adolescents aged 16–18 years. Eur Child Adolesc Psychiatry. 2014;23(8):681-9.
55. Blom K. Guided internet-based treatment for insomnia and depression. In: Institutionen för Klinisk N, editor. Stockholm: Inst för klinisk neurovetenskap / Dept of Clinical Neuroscience; 2016.
56. Depression in children and young people: identification and management. National Institute for Health and Care Excellence; 2005. Report No.: CG28.
57. Elizabeth McCauley KAS, Gretchen R. Gudmundsen, Christopher R. Martell, and Sona Dimidjian. Behavioral Activation with Adolescents

A Clinician's Guide: Guilford press; 2016.
58. Webster-Stratton C, Herman KC. The Impact of Parent Behavior-Management Training on Child Depressive Symptoms. J Couns Psychol. 2008;55(4):473-84.
59. Wyatt Kaminski J, Valle L, Filene J, Boyle C. A Meta-analytic Review of Components Associated with Parent Training Program Effectiveness. J Abnorm Child Psychol. 2008;36(4):567-89.
60. Ioannidis JP, Evans SJ, Gotzsche PC, O'Neill RT, Altman DG, Schulz K, et al. Better reporting of harms in randomized trials: an extension of the CONSORT statement. Annals of internal medicine. 2004;141(10):781-8.
61. Topooco N, Berg M, Johansson S, Liljethörn L, Radvogin E, Vlaescu G, et al. Chat- and internet-based cognitive-behavioural therapy in treatment of adolescent depression: randomised controlled trial. BJPsych open. 2018;4(4):199.
62. Andrén P, Aspvall K, Fernández de la Cruz L, Wiktor P, Romano S, Andersson E, et al. Therapist-guided and parent-guided internet-delivered behaviour therapy for paediatric Tourette's disorder: a pilot randomised controlled trial with long-term follow-up. BMJ Open. 2019;9(2):e024685.

Appendix 1. Original power calculation

Due to a miscommunication with the trial statistician, the original power calculation was erroneously based on measurements at time points 0, 3 and 12 months, and did not include data attrition. The updated power calculation in version 1.5. of this study protocol (performed in September 2023), is based on measurements at time points 0, 3 and 6 months, and attrition is included in the analysis. The power calculation was updated without looking at data, and thus the outcome assessors and trial statistician remain blinded.

The original power calculation was as follows:

We estimated the statistical power for different sample sizes and between-group difference for the test of the joint null hypothesis that the two experimental groups (guided and self-guided) are both equal to the TAU group to detect a difference between each experimental group and TAU at three months follow-up of six points in CDRS-R. A 6-point difference on the primary outcome measure was the minimal change that could be considered as clinically meaningful according to three psychologists with experience of using the CDRS-R. Taking attrition into account, a sample size of N=225 would provide an >80% power of detecting a six-point difference. The results from the conducted pilot study indicates that an effect of at least this size can be expected between the guided I-BA and self-guided I-BA compared with TAU.

Signature page

This document has been electronically signed using eduSign.

eduSign

Electronically signed by
Eva Serlachius

Date and time of signature
2023-11-02 08:59 UTC

Authenticated by
Karolinska Institutet

Validate signed documents at: https://validator.edusign.sunet.se

SUNET
