# Supplementary material for: Single-blinded, randomised, parallel-group, controlled trial comparing the efficacy and cost-effectiveness of therapist- and self-guided internet-delivered behavioural activation versus treatment as usual for adolescents with mild to moderate depression: study protocol
Source: BMJ Open. 2024 Oct 15;14(10):e083507. doi: 10.1136/bmjopen-2023-083507 (PMC11590840; doi:10.1136/bmjopen-2023-083507)

Supplemental file 2. Screenshots of the I-BA interventions

1. Left: Psychoeducational video on depression. Right: Activity diary used throughout treatment.

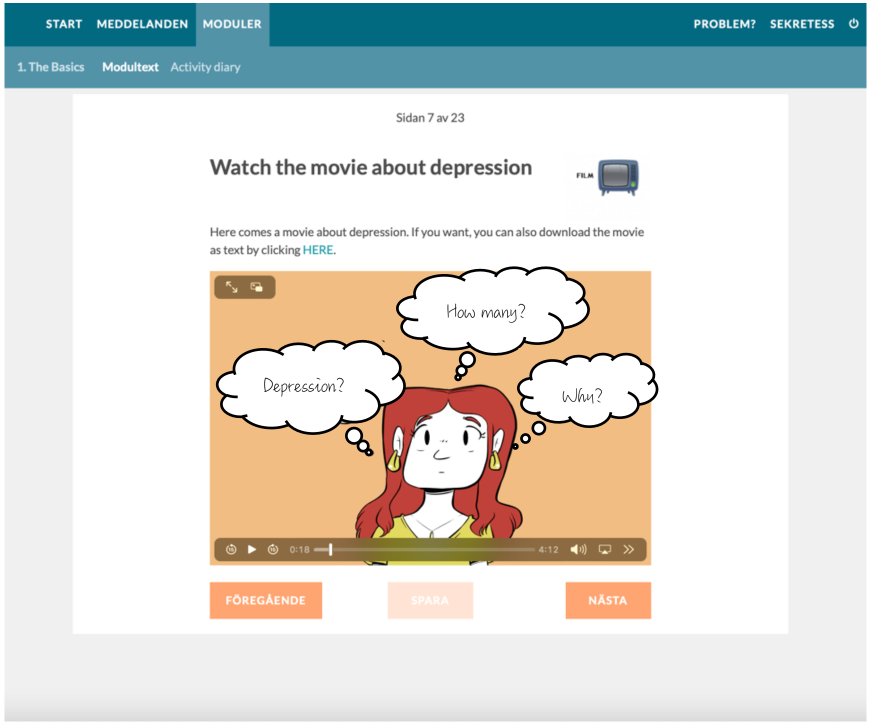

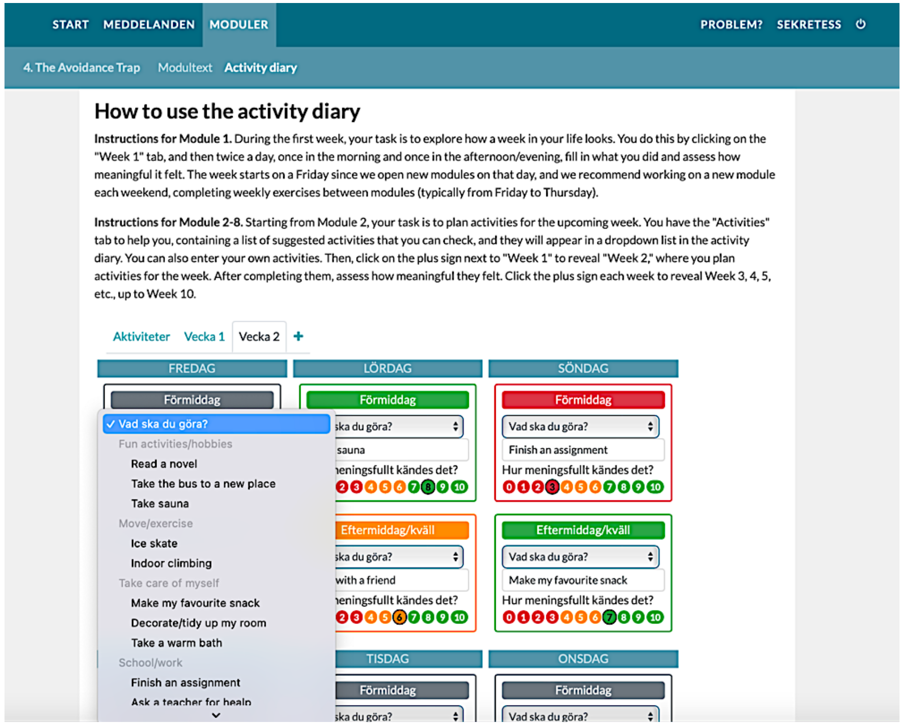

2. Left: The three fictional characters sharing their treatment goals. Right: Exercise on the avoidance trap.

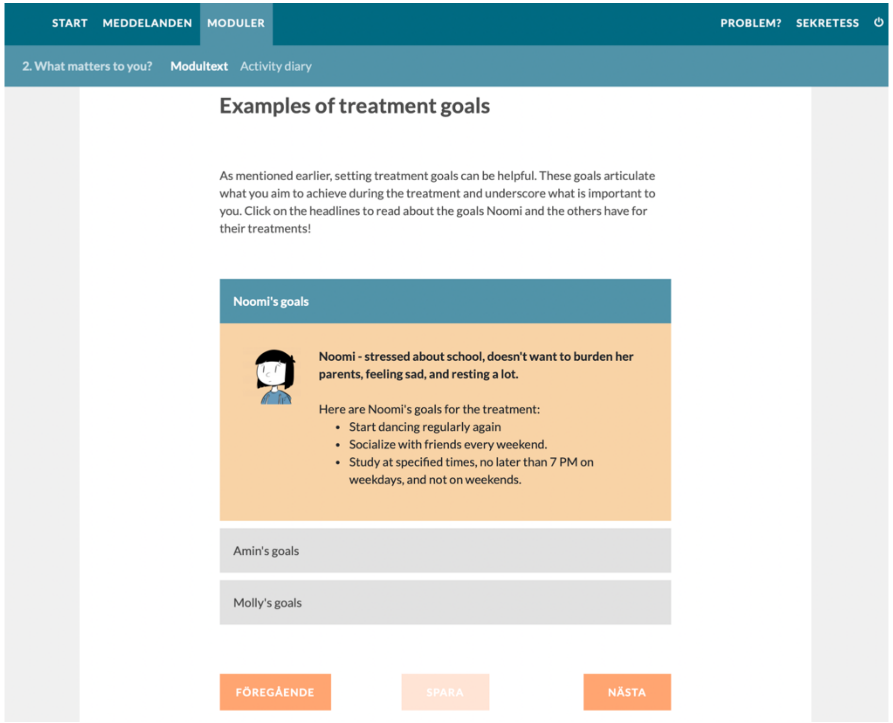

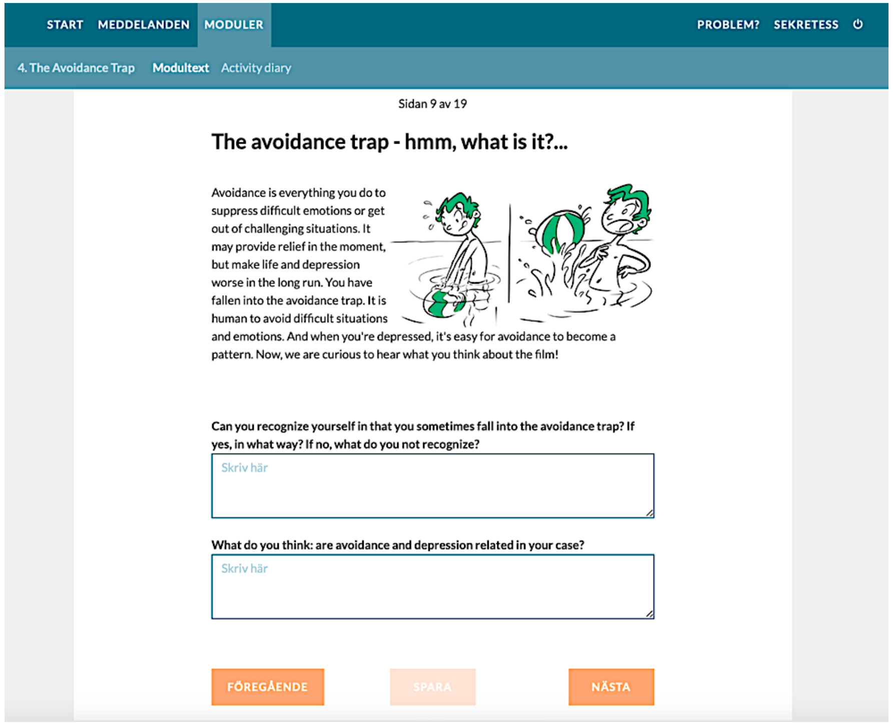

3. Left: Psychoeducation about common parent traps. Right: Information on validating one's adolescent's feelings.

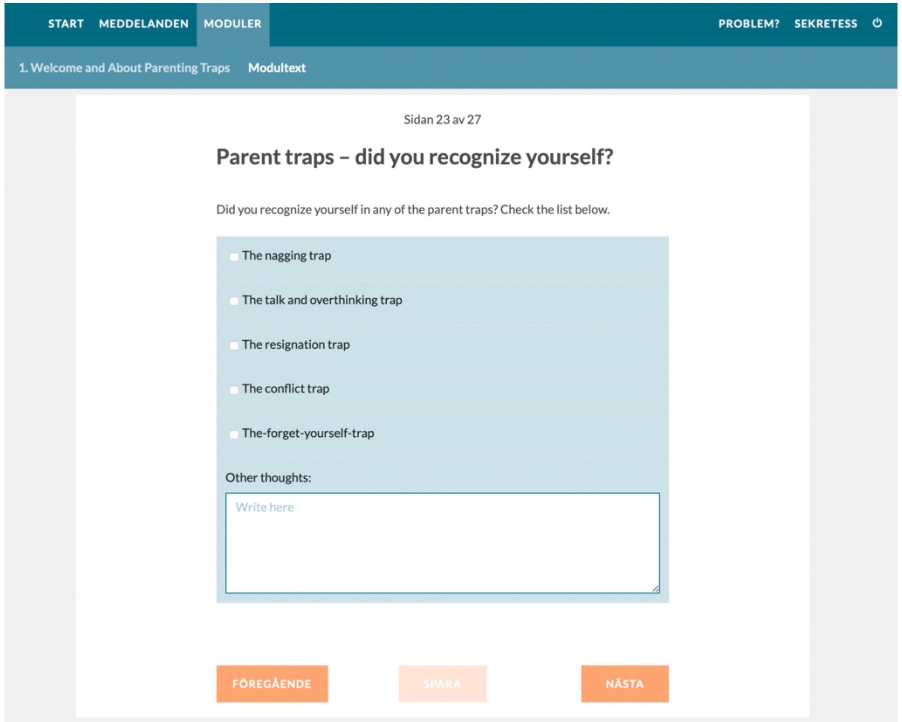

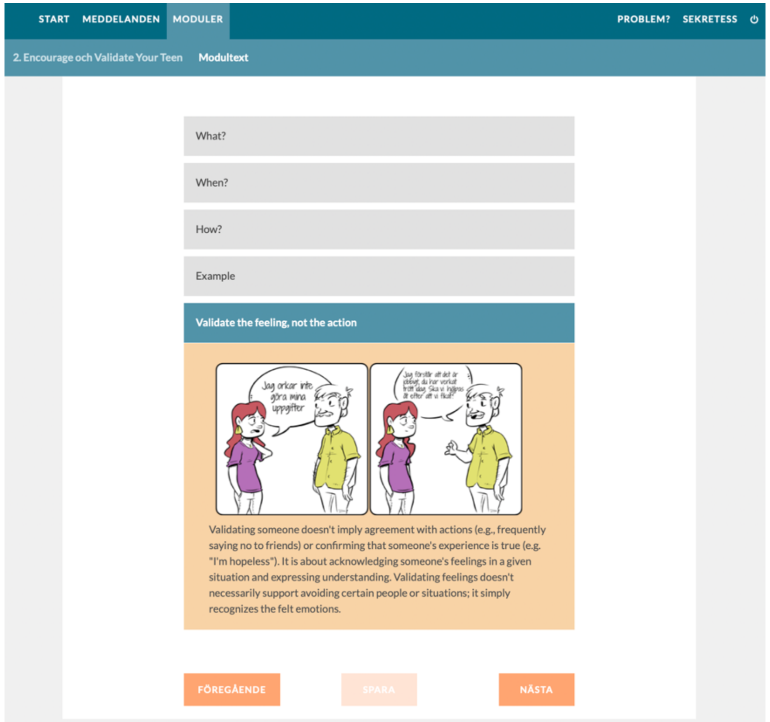

4. Guided I-BA includes an encrypted messaging feature. Psychologist will respond within 1-2 working days. Example below with a fictitious patient.

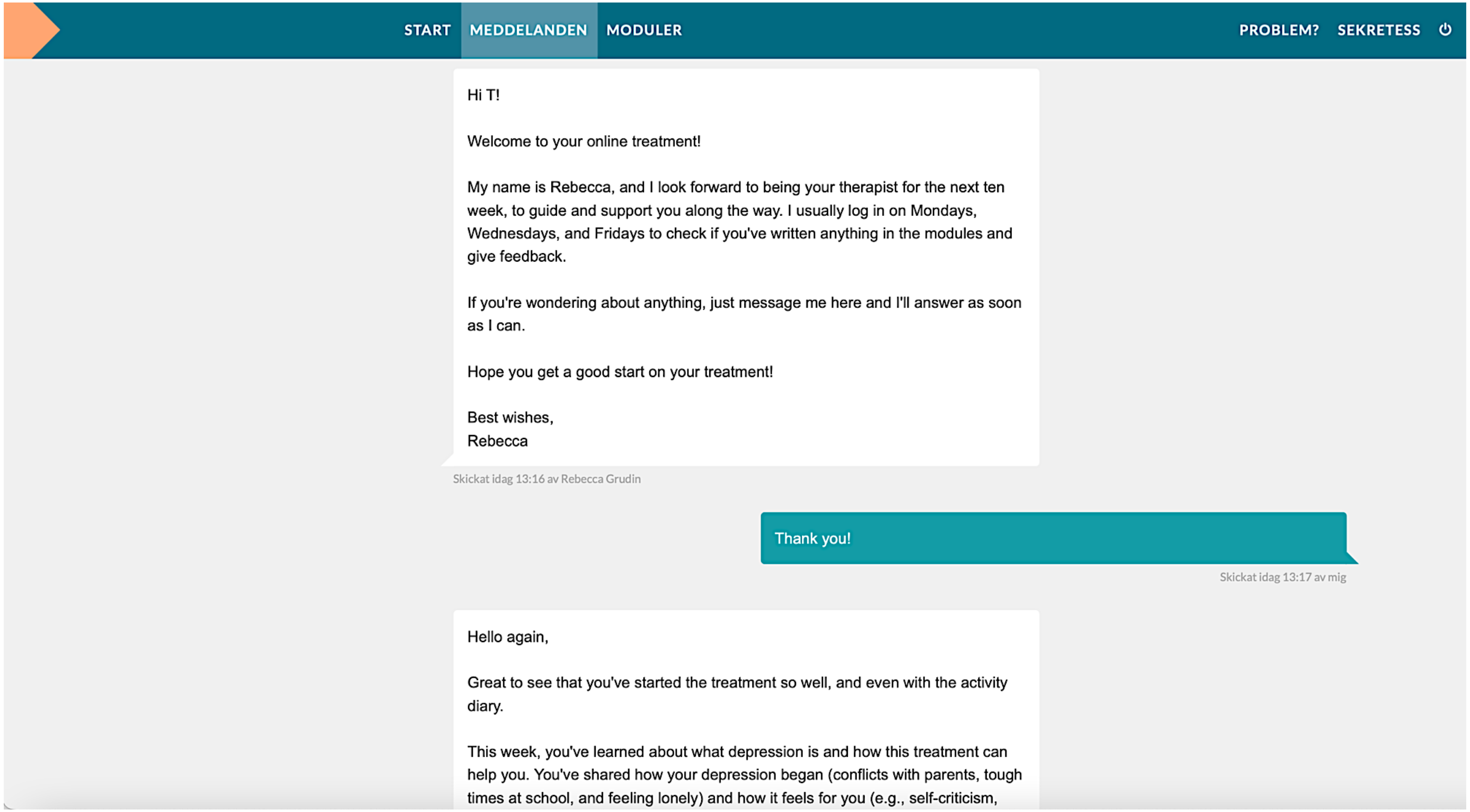

Supplement: online supplemental file 2 [file bmjopen-14-10-s002.pdf]
