# Supplementary material for: Single-blinded, randomised, parallel-group, controlled trial comparing the efficacy and cost-effectiveness of therapist- and self-guided internet-delivered behavioural activation versus treatment as usual for adolescents with mild to moderate depression: study protocol
Source: BMJ Open. 2024 Oct 15;14(10):e083507. doi: 10.1136/bmjopen-2023-083507 (PMC11590840; doi:10.1136/bmjopen-2023-083507)
Supplement: Supplementary file 3 [file bmjopen-2023-083507.reviewer_comments.pdf]

PEER REVIEW HISTORY

BMJ Open publishes all reviews undertaken for accepted manuscripts. Reviewers are asked to complete a checklist review form and are provided with free text boxes to elaborate on their assessment. These free text comments are reproduced below.

ARTICLE DETAILS

Title (Provisional)

Study protocol for a single-blinded, randomised, parallel-group, controlled trial comparing the efficacy and cost-effectiveness of therapist- and self-guided internet-delivered behavioural activation versus treatment as usual for adolescents with mild to moderate depression

Authors

Andersson, Rebecca; Vigerland, Sarah; Lenhard, Fabian; Ahlen, Johan; Bottai, Matteo; Mataix-Cols, David; SERLACHIUS, Eva

---

VERSION 1 - REVIEW

**Reviewer**	**1**
**Name**	**Loades, Maria**
**Affiliation**	**University of Bath, Psychology**
**Date**	**13-Mar-2024**
**COI**	**None identified**

This is an interesting trial, well described in this protocol and will certainly be a valuable addition. Just a few things to consider/amend:

- title and thoroughout, is this efficacy or effectiveness?

- introduction - I suggest moving the latter part of paragraph 1 where you introduce digital treatments to the beginning of para 2. Also please add to para 3 references to reviews showing that effects are larger in tx guided vs self help digital txs in adolescents too - e.g. Grist et al, Pennant et al, Lehtimaki et al. Also please add a line in the introduction to say which type of BA you are referring to - lejuez et al or Martell et al? I think it is Lejuez - values based BA, also called BATD?

-method - it may be clearer to refer to clinician rated as assessor rated, as the clinician is the treating psychologist who I don’t think is the rater? Also please describe in more detail who the clinicians are, what training and supervision they have for this work. Please add the number of items on the RCADS-anxiety where you describe this.

**Reviewer**	**2**
**Name**	**Lindqvist, Karin**

**Affiliation**	**Stockholm University, Department of Psychologyq**
**Date**	**22-Mar-2024**
**COI**	**None**

This is a very well written and ambitious research protocol. The trial is well planned and well described. It is easy to follow the planned procedures and the rational for them. I only have a few minor comments.

1. P 12. It is not clear whether amount of phone calls will be logged (and presented in the outcome paper). I see that the authors state that therapist time will be logged, but it is of interest how much of this time is spent on phone calls, since this is an internet-delivered text-based treatment. Ideally the number of and time spent on phone calls should be presented separately.

2. P 14. In your SPIRIT schedule, it is stated that only parents fill in TIC-P. Since you have participants up to 17, it is possible that they are seeking healthcare without parent's knowledge. This should be discussed.

3. You state that your response measure will be the CGI-I. Why will you not use the primary outcome measure as a measure of response? Furthermore, would it not make sense to also calculate response/remission on a self-rated measure, such as using clinical cut-offs on QIDS or calculating the RCI – even if CGI-I is the primary measure? That would make for an interesting comparison and increase robustness, if observer-rated and self-rated numbers of response/remission correspond.

4. You state that the primary endpoint is 3 months post-treatment, and provide a rationale for this. However, it is not clear to me whether you will also report results at post-treatment (your secondary analyses only states 12 months F-U). This should be included.

5. You state that a standardized effect size of 0.5 aligns with your clinical impression of a minimal clinically significant change. However, some would argue that much smaller changes may be clinically meaningful. For instance, Cuijpers et al., (2014) suggest that SMDs as small as 0.24 may be clinically relevant. What is your perspective on that, since, if adhering to these suggested clinically relevant effects, it could mean that your trial may be underpowered to detect meaningful differences? I think this needs to be discussed not only on basis on clinical impression but also on basis on previous research and other suggested MCDs.

I look forward to following this interesting trial and taking part of the results! Best of luck.

VERSION 1 - AUTHOR RESPONSE

Reviewer: 1

Dr. Maria Loades, University of Bath, University of Bristol School of Social and Community Medicine

Comments to the Author:

This is an interesting trial, well described in this protocol and will certainly be a valuable addition. Just a few things to consider/amend:

- Title and throughout, is this efficacy or effectiveness?

Thank you for an interesting question! We consider this an efficacy trial because of the high level of control and numerous measures aimed at reducing bias, such as blinded follow-ups, well-trained staff, and significant recruitment efforts. But there are also elements of pragmatic design present. For instance, the trial setting is like the regular internet treatment unit within CAMHS in Stockholm, TAU is provided by standard care, which is heterogeneous in terms of provided interventions. In addition, medication is permitted during the study if it is stable. We have assessed our trial using the PRECIS-2 tool (Dal-Ré, Janiaud & Ioannidis, 2018), which indicates a clear weighting towards an efficacy trial rather than a pragmatic trial.

It may be confusing that we use the term cost-effectiveness in the title and throughout the paper, but this is the conventional term used in the field of health economic evaluation for the specific analyses we intend to carry out, so we have not changed this term.

Introduction

- I suggest moving the latter part of paragraph 1 where you introduce digital treatments to the beginning of para 2.

Thank you for these suggestions. We have changed accordingly.

Also please add to para 3 references to reviews showing that effects are larger in tx guided vs self-help digital txs in adolescents too – e.g. Grist et al, Pennant et al, Lehtimaki et al.

We have added Grist et al 2019 and Pennant et al 2015 and updated this part in the Introduction on page 4.

Also please add a line in the introduction to say which type of BA you are referring to - lejuez et al or Martell et al? I think it is Lejuez - values based BA, also called BATD?

We don't believe we can classify the experimental intervention as either BATD or Martell's BA since it is a mixture of the elements of both protocols, tailored for internet delivery. Like BATD, it's concise (8 modules) and contains values. Like Martell's BA model, it addresses avoidance, but does not include formal functional analysis. We provide more detail about the intervention in the Method section of the paper, where we also provide references to both versions of BA (pages 9-11).

-method - it may be clearer to refer to clinician rated as assessor rated, as the clinician is the treating psychologist who I don't think is the rater?

Thank you for this suggestion. "Clinician rated" has been changed to "(blinded) assessor rated" throughout the manuscript. For the sake of clarity, we have also replaced "psychologist" with "therapist" in the section on the I-BA interventions (pages 9-10).

Also please describe in more detail who the clinicians are, what training and supervision they have for this work.

We have added the following information: "Therapists in the trial must be licensed psychologists with CBT training, and specific training to deliver the I-BA intervention. All therapists engage in weekly peer supervision sessions." (page 10).

Regarding training of blinded assessors, we added this information on page 9:

"Blinded assessors consist of three licensed psychologists and three psychology students (completed the first three years of the psychology program). The students have continuous access to senior colleagues for guidance and support."

Please add the number of items on the RCADS-anxiety where you describe this.

Information about number of items has been added (page 13).

Reviewer: 2

Dr. Karin Lindqvist, Stockholm University

Comments to the Author:

This is a very well written and ambitious research protocol. The trial is well planned and well described. It is easy to follow the planned procedures and the rational for them. I only have a few minor comments.

1. P 12. It is not clear whether amount of phone calls will be logged (and presented in the outcome paper). I see that the authors state that therapist time will be logged, but it is of interest how much of this time is spent on phone calls, since this is an internet-delivered text-based treatment. Ideally the number of and time spent on phone calls should be presented separately.

Thank you for pointing this out. We have added information on page 12 that phone call data will be presented as part of therapist time as well as separately.

2. P 14. In your SPIRIT schedule, it is stated that only parents fill in TIC-P. Since you have participants up to 17, it is possible that they are seeking healthcare without parent's knowledge. This should be discussed.

We agree that this is a limitation. However, it was a choice we made to spare participants from filling out more questionnaires. Given the sample size, any biases should be distributed equally between the groups and not influence the relative differences in costs.

We also collect data on co-occurring treatments from the adolescents. These data will not be used in the health economic evaluations but will allow us to explore potential differences between parent and adolescent reporting on patterns of care-seeking behaviours.

3. You state that your response measure will be the CGI-I. Why will you not use the primary outcome measure as a measure of response? Furthermore, would it not make sense to also calculate response/remission on a self-rated measure, such as using clinical cut-offs on QIDS or calculating the RCI – even if CGI-I is the primary measure? That would make for an interesting comparison and increase robustness, if observer-rated and self-rated numbers of response/remission correspond.

CGI-I is a response measure commonly used in intervention trials for adolescents with depression (e.g., Mufson et al, 2004, TADS 2003, McCauley et al 2015). We could have used a combined score of the CDRS-R and the CGI-I, but we choose the CGI-I. One reason was that there is no Swedish validation for CDRS-R, therefore difficult to choose a valid cut-off that corresponds to clinical improvement.

4. You state that the primary endpoint is 3 months post-treatment and provide a rationale for this. However, it is not clear to me whether you will also report results at post-treatment (your secondary analyses only states 12 months FU). This should be included.

Thank you for the opportunity to clarify this. We will not run any analyses and test for changes between pre-treatment and post-treatment, as the latter is not our prespecified primary endpoint. However, will include post-treatment data in the regression models, which will increase statistical power. The post-treatment data will also be presented in a table in the Results for descriptive purposes.

5. You state that a standardized effect size of 0.5 aligns with your clinical impression of a minimal clinically significant change. However, some would argue that much smaller changes may be clinically meaningful. For instance, Cuijpers et al., (2014) suggest that SMDs as small as 0.24 may be clinically relevant. What is your perspective on that, since, if adhering to these suggested clinically relevant effects, it could mean that your trial may be underpowered to detect meaningful differences? I think this needs to be discussed not only on basis on clinical impression but also on basis on previous research and other suggested MCDs.

Thank you for bringing up this interesting article! During our power calculation, our statistician recommended that we consider the between group difference in raw scores on our primary outcome (CDRS-R, range 17-113) rather than focusing solely on a specific effect size. While we didn't directly consult patients, akin to the approach described by Cuijiper et al. 2014, we embraced the notion that effect size alone may not adequately capture clinically relevant differences, as this depends on the nature of the outcome measure itself. Therefore, we considered the magnitude of the between-group difference in CDRS-R scores that might matter in a patient's life (6 points), along with looking at other influential trials on adolescent depression. When we converted the raw scores to SMD, we arrived at 0.5, a common threshold for assessing superiority in clinical trials.

Looking at the broader literature, different authors have taken different approaches. Some studies made similar choices to ours (see for example Merry et al 2012, ICBT vs TAU, which used a 5.5-point non-inferior margin, i.e. indicating that in a superior trial one should not be below 5.5). For others a smaller effect size (0.15) than ours (0.50) was used in their power calculation (see for example Richardson et al 2014, comparing collaborative care vs usual care).

Yoshimatsu et al. 2023 consider as much as a 14-point change on the CDRS-R as a clinically relevant change (defined as a CGI-I score of 3, i.e., minimally improved). The authors emphasize that this is not, however, a difference one would typically expect between groups. The discussion concludes: “However, it seems to be difficult to expect a 14-point treatment

difference of the CDRS-R for antidepressants compared with that with placebo, [...] Based on our findings, the magnitude of the treatment difference for fluoxetine and fluoxetine with cognitive-behavioral therapy compared with that for placebo was half or more of the CDRS-R improvement, corresponding to minimal improvement in the CGI [our emphasis].” Given the latter, the smallest clinically relevant drop in CDRS-R that we have chosen (6 points) is close to what Yoshimatsu et al claim (7 points).

To sum this long answer (!), there are many different perspectives to consider, and there does not seem to be one true answer. We will interpret the results of our study in relation to the methodological choices we made.

VERSION 2 - REVIEW

**Reviewer**	**1**
**Name**	**Loades, Maria**
**Affiliation**	**University of Bath, Psychology**
**Date**	**09-May-2024**
**COI**	**None identified**

Thank you for your clear and carefully considered responses.

**Reviewer**	**2**
**Name**	**Lindqvist, Karin**
**Affiliation**	**Stockholm University, Department of Psychologyq**
**Date**	**23-May-2024**
**COI**	**None**

Comments to the Author:

This is a very well written and ambitious research protocol. The trial is well planned and well described. It is easy to follow the planned procedures and the rational for them. I only have a few minor comments.

1. P 12. It is not clear whether amount of phone calls will be logged (and presented in the outcome paper). I see that the authors state that therapist time will be logged, but it is of interest how much of this time is spent on phone calls, since this is an internet-delivered text-based treatment. Ideally the number of and time spent on phone calls should be presented separately.

Thank you for pointing this out. We have added information on page 12 that phone call data will be presented as part of therapist time as well as separately.

2. P 14. In your SPIRIT schedule, it is stated that only parents fill in TIC-P. Since you have participants up to 17, it is possible that they are seeking healthcare without parent's knowledge. This should be discussed.

We agree that this is a limitation. However, it was a choice we made to spare participants from filling out more questionnaires. Given the sample size, any biases should be distributed equally between the groups and not influence the relative differences in costs.

We also collect data on co-occurring treatments from the adolescents. These data will not be used in the health economic evaluations but will allow us to explore potential differences between parent and adolescent reporting on patterns of care-seeking behaviours.

3. You state that your response measure will be the CGI-I. Why will you not use the primary outcome measure as a measure of response? Furthermore, would it not make sense to also calculate response/remission on a self-rated measure, such as using clinical cut-offs on QIDS or calculating the RCI – even if CGI-I is the primary measure? That would make for an interesting comparison and increase robustness, if observer-rated and self-rated numbers of response/remission correspond.

CGI-I is a response measure commonly used in intervention trials for adolescents with depression (e.g., Mufson et al, 2004, TADS 2003, McCauley et al 2015). We could have used a combined score of the CDRS-R and the CGI-I, but we choose the CGI-I. One reason was that there is no Swedish validation for CDRS-R, therefore difficult to choose a valid cut-off that corresponds to clinical improvement.

4. You state that the primary endpoint is 3 months post-treatment and provide a rationale for this. However, it is not clear to me whether you will also report results at post-treatment (your secondary analyses only states 12 months FU). This should be included.

Thank you for the opportunity to clarify this. We will not run any analyses and test for changes between pre-treatment and post-treatment, as the latter is not our prespecified primary endpoint. However, will include post-treatment data in the regression models, which will increase statistical power. The post-treatment data will also be presented in a table in the Results for descriptive purposes.

5. You state that a standardized effect size of 0.5 aligns with your clinical impression of a minimal clinically significant change. However, some would argue that much smaller changes may be clinically meaningful. For instance, Cuijpers et al., (2014) suggest that SMDs as small as 0.24 may be clinically relevant. What is your perspective on that, since, if adhering to these suggested clinically relevant effects, it could mean that your trial may be underpowered to detect meaningful differences? I think this needs to be discussed not only on basis on clinical impression but also on basis on previous research and other suggested MCDs.

Thank you for bringing up this interesting article! During our power calculation, our statistician recommended that we consider the between group difference in raw scores on our primary outcome (CDRS-R, range 17-113) rather than focusing solely on a specific effect size. While we didn't directly consult patients, akin to the approach described by Cuijiper et al. 2014, we embraced the notion that effect size alone may not adequately capture clinically relevant

differences, as this depends on the nature of the outcome measure itself. Therefore, we considered the magnitude of the between-group difference in CDRS-R scores that might matter in a patient's life (6 points), along with looking at other influential trials on adolescent depression. When we converted the raw scores to SMD, we arrived at 0.5, a common threshold for assessing superiority in clinical trials.

Looking at the broader literature, different authors have taken different approaches. Some studies made similar choices to ours (see for example Merry et al 2012, ICBT vs TAU, which used a 5.5-point non-inferior margin, i.e. indicating that in a superior trial one should not be below 5.5). For others a smaller effect size (0.15) than ours (0.50) was used in their power calculation (see for example Richardson et al 2014, comparing collaborative care vs usual care).

Yoshimatsu et al. 2023 consider as much as a 14-point change on the CDRS-R as a clinically relevant change (defined as a CGI-I score of 3, i.e., minimally improved). The authors emphasize that this is not, however, a difference one would typically expect between groups. The discussion concludes: "However, it seems to be difficult to expect a 14-point treatment difference of the CDRS-R for antidepressants compared with that with placebo, […] Based on our findings, the magnitude of the treatment difference for fluoxetine and fluoxetine with cognitive-behavioral therapy compared with that for placebo was half or more of the CDRS-R improvement, corresponding to minimal improvement in the CGI [our emphasis]." Given the latter, the smallest clinically relevant drop in CDRS-R that we have chosen (6 points) is close to what Yoshimatsu et al claim (7 points).

To sum this long answer (!), there are many different perspectives to consider, and there does not seem to be one true answer. We will interpret the results of our study in relation to the methodological choices we made.

Reviewer: 2
Dr. Karin Lindqvist, Stockholm University

Comments to the Author:
This is a very well written and ambitious research protocol. The trial is well planned and well described. It is easy to follow the planned procedures and the rational for them. I only have a few minor comments.

1. P 12. It is not clear whether amount of phone calls will be logged (and presented in the outcome paper). I see that the authors state that therapist time will be logged, but it is of interest how much of this time is spent on phone calls, since this is an internet-delivered text-based treatment. Ideally the number of and time spent on phone calls should be presented separately.
Thank you for pointing this out. We have added information on page 12 that phone call data will be presented as part of therapist time as well as separately.

This is perfect!

2. P 14. In your SPIRIT schedule, it is stated that only parents fill in TIC-P. Since you have participants up to 17, it is possible that they are seeking healthcare without parent's knowledge. This should be discussed.
We agree that this is a limitation. However, it was a choice we made to spare participants from filling out more questionnaires. Given the sample size, any biases should be distributed equally between the groups and not influence the relative differences in costs.

We also collect data on co-occurring treatments from the adolescents. These data will not be used in the health economic evaluations but will allow us to explore potential differences between parent and adolescent reporting on patterns of care-seeking behaviours.

This makes sense and sounds well thought through. I do however think this could be mentioned in the manuscript.

3. You state that your response measure will be the CGI-I. Why will you not use the primary outcome measure as a measure of response? Furthermore, would it not make sense to also calculate response/remission on a self-rated measure, such as using clinical cut-offs on QIDS or calculating the RCI – even if CGI-I is the primary measure? That would make for an interesting comparison and increase robustness, if observer-rated and self-rated numbers of response/remission correspond.
CGI-I is a response measure commonly used in intervention trials for adolescents with depression (e.g., Mufson et al, 2004, TADS 2003, McCauley et al 2015). We could have used a combined score of the CDRS-R and the CGI-I, but we choose the CGI-I. One reason was that there is no Swedish validation for CDRS-R, therefore difficult to choose a valid cut-off that corresponds to clinical improvement.

To clarify: my suggestion was not to not use the CGI-I, but to add an additional measure of response/remission, since you have both observer-rated and self-rated measures. I am aware that the CGI is an established measure but it would also make sense to, in addition, calculate response/remission on your primary measure. As I said, you do not need to use validated cut-off-scores, you could calculate the RCI, which is also a very commonly used way of calculating response/remission.

4. You state that the primary endpoint is 3 months post-treatment and provide a rationale for this. However, it is not clear to me whether you will also report results at post-treatment (your secondary analyses only states 12 months FU). This should be included.
Thank you for the opportunity to clarify this. We will not run any analyses and test for changes between pre-treatment and post-treatment, as the latter is not our prespecified primary endpoint. However, will include post-treatment data in the regression models, which will increase statistical power. The post-treatment data will also be presented in a table in the Results for descriptive purposes.

I must admit that I do not understand why you don't plan on presenting comparison data from your post-treatment-assessment, even if your primary endpoint is 3 months. This is very uncommon in psychotherapy outcome trials, I do understand that you probably have a sound rationale for this (one being that you believe that more patients in TAU have received treatment by then, but that's not really the point of follow-up measurements, I would say that the assumption must be that patients in a clinical trial are treated during the treatment period), but personally I become very curious about this, and would like to see a further discussion about this.

5. You state that a standardized effect size of 0.5 aligns with your clinical impression of a minimal clinically significant change. However, some would argue that much smaller changes may be clinically meaningful. For instance, Cuijpers et al., (2014) suggest that SMDs as small as 0.24 may be clinically relevant. What is your perspective on that, since, if adhering to these suggested clinically relevant effects, it could mean that your trial may be underpowered to detect meaningful differences? I think this needs to be discussed not only on basis on clinical impression but also on basis on previous research and other suggested MCDs.

Thank you for bringing up this interesting article! During our power calculation, our statistician recommended that we consider the between group difference in raw scores on our primary outcome (CDRS-R, range 17-113) rather than focusing solely on a specific effect size. While we didn't directly consult patients, akin to the approach described by Cuijiper et al. 2014, we embraced the notion that effect size alone may not adequately capture clinically relevant differences, as this depends on the nature of the outcome measure itself. Therefore, we considered the magnitude of the between-group difference in CDRS-R scores that might matter in a patient's life (6 points), along with looking at other influential trials on adolescent depression. When we converted the raw scores to SMD, we arrived at 0.5, a common threshold for assessing superiority in clinical trials.

Looking at the broader literature, different authors have taken different approaches. Some studies made similar choices to ours (see for example Merry et al 2012, ICBT vs TAU, which used a 5.5-point non-inferior margin, i.e. indicating that in a superior trial one should not be below 5.5). For others a smaller effect size (0.15) than ours (0.50) was used in their power calculation (see for example Richardson et al 2014, comparing collaborative care vs usual care).

Yoshimatsu et al. 2023 consider as much as a 14-point change on the CDRS-R as a clinically relevant change (defined as a CGI-I score of 3, i.e., minimally improved). The authors emphasize that this is not, however, a difference one would typically expect *between* groups. The discussion concludes: "However, it seems to be difficult to expect a 14-point treatment difference of the CDRS-R for antidepressants compared with that with placebo, […] Based on our findings, the magnitude of the treatment difference for fluoxetine and fluoxetine with cognitive-behavioral therapy compared with that for placebo was *half or more of the CDRS-R improvement, corresponding to minimal improvement in the CGI* [our emphasis]." Given the latter, the smallest clinically relevant drop in CDRS-R that we have chosen (6 points) is close to what Yoshimatsu et al claim (7 points).

To sum this long answer (!), there are many different perspectives to consider, and there does not seem to be one true answer. We will interpret the results of our study in relation to the methodological choices we made.

Thank you for this thorough answer! I completely agree that there does not seem to be one true answer when it comes to thresholds for clinically relevant change, and I think you show this and discuss it very well in this response. Why not include some of this discussion in the article? In my opinion, since it is such a difficult definition, a short discussion in the article of your choice versus other possible choices would be very interesting!

VERSION 2 - AUTHOR RESPONSE

Response to editor and reviewers' comments, 2024-08-23

Note that reviewers' comments are in italics. All page references below are to the marked version of the manuscript.

Reviewer 1
Dr. Maria Loades, University of Bath, University of Bristol School of Social and Community Medicine

Comments to the Author: *Thank you for your clear and carefully considered responses.*

Thank you for your kind feedback. We're glad that the revisions have satisfied your concerns.

Reviewer 2
Dr. Karin Lindqvist, Stockholm University
Comments to the Author: *This is a very well written and ambitious research protocol. The trial is well planned and well described. It is easy to follow the planned procedures and the rational for them. I only have a few minor comments.*

1. P 12. It is not clear whether amount of phone calls will be logged (and presented in the outcome paper). I see that the authors state that therapist time will be logged, but it is of interest how much of this time is spent on phone calls, since this is an internet-delivered text-based treatment. Ideally the number of and time spent on phone calls should be presented separately.

Thank you for pointing this out. We have added information on page 12 that phone call data will be presented as part of therapist time as well as separately.

This is perfect!

2. P 14. In your SPIRIT schedule, it is stated that only parents fill in TIC-P. Since you have participants up to 17, it is possible that they are seeking healthcare without parent's knowledge. This should be discussed.

We agree that this is a limitation. However, it was a choice we made to spare participants from filling out more questionnaires. Given the sample size, any biases should be distributed equally between the groups and not influence the relative differences in costs.

We also collect data on co-occurring treatments from the adolescents. These data will not be used in the health economic evaluations but will allow us to explore potential differences between parent and adolescent reporting on patterns of care-seeking behaviours.

This makes sense and sounds well thought through. I do however think this could be mentioned in the manuscript.

Thank you for your suggestion. We have added this information to the manuscript on page 13 (lines 30-33) and page 14 (lines 1-6). Additionally, the TiC-P is not administered to adolescents because questions about parents' work, productivity loss, and childcare are not applicable to them.

3. You state that your response measure will be the CGI-I. Why will you not use the primary outcome measure as a measure of response? Furthermore, would it not make sense to also calculate response/remission on a self-rated measure, such as using clinical cut-offs on QIDS or calculating the RCI – even if CGI-I is the primary measure? That would make for an interesting comparison and increase robustness, if observer-rated and self-rated numbers of response/remission correspond.

CGI-I is a response measure commonly used in intervention trials for adolescents with depression (e.g., Mufson et al, 2004, TADS 2003, McCauley et al 2015). We could have used a combined score of the CDRS-R and the CGI-I, but we choose the CGI-I. One reason was that there is no Swedish validation for CDRS-R, therefore difficult to choose

a valid cut-off that corresponds to clinical improvement.

To clarify: my suggestion was not to not use the CGI-I, but to add an additional measure of response/remission, since you have both observer-rated and self-rated measures. I am aware that the CGI is an established measure, but it would also make sense to, in addition, calculate response/remission on your primary measure. As I said, you do not need to use validated cut-off-scores, you could calculate the RCI, which is also a very commonly used way of calculating response/remission.

This is an interesting suggestion. However, we prefer to use the CGI-I, a decision made a priori, i.e., before the trial started. Also, we want to avoid the problem of using multiple definitions of response/remission, which can create confusion for the reader because you don't know which one is most correct. Thus, we prefer to adhere to the original analysis plan.

4. You state that the primary endpoint is 3 months post-treatment and provide a rationale for this. However, it is not clear to me whether you will also report results at post-treatment (your secondary analyses only state 12 months FU). This should be included.

Thank you for the opportunity to clarify this. We will not run any analyses and test for changes between pre-treatment and post-treatment, as the latter is not our prespecified primary endpoint. However, we will include post-treatment data in the regression models, which will increase statistical power. The post-treatment data will also be presented in a table in the Results for descriptive purposes.

I must admit that I do not understand why you don't plan on presenting comparison data from your post-treatment-assessment, even if your primary endpoint is 3 months. This is very uncommon in psychotherapy outcome trials, I do understand that you probably have a sound rationale for this (one being that you believe that more patients in TAU have received treatment by then, but that's not really the point of follow-up measurements, I would say that the assumption must be that patients in a clinical trial are treated during the treatment period), but personally I become very curious about this, and would like to see a further discussion about it.

Aside from the argument that the 3FU provides a fair comparison between our experimental and comparison groups, instead of favoring the experimental groups, presenting post-data descriptively only may help avoid issues with multiple testing and Type I error rates. We prefer to adhere to the original analysis plan.

5. You state that a standardized effect size of 0.5 aligns with your clinical impression of a minimal clinically significant change. However, some would argue that much smaller changes may be clinically meaningful. For instance, Cuijpers et al., (2014) suggest that SMDs as small as 0.24 may be clinically relevant. What is your perspective on that, since, if adhering to these suggested clinically relevant effects, it could mean that your trial may be underpowered to detect meaningful differences? I think this needs to be discussed not only on basis on clinical impression but also on basis on previous research and other suggested MCDs.

Thank you for bringing up this interesting article! During our power calculation, our statistician recommended that we consider the between group difference in raw scores on our primary outcome (CDRS-R, range 17-113) rather than focusing solely on a specific effect size. While we didn't directly consult patients, akin to the approach described by Cuijiper et al. 2014, we embraced the notion that effect size alone may not adequately capture clinically relevant differences, as this depends on the nature of the outcome measure itself. Therefore, we considered the magnitude of the between-group difference in CDRS-R scores that might matter in a patient's life (6 points), along with looking at other influential trials on adolescent depression. When we converted the raw scores to SMD, we arrived at 0.5, a common threshold for assessing superiority in clinical trials.

Looking at the broader literature, different authors have taken different approaches. Some studies made similar choices to ours (see for example Merry et al 2012, ICBT vs TAU, which used a 5.5-point non-inferior margin, i.e. indicating that in a superior trial one should not be below 5.5). For others a smaller effect size (0.15) than ours (0.50) was used in their power calculation (see for example Richardson et al 2014, comparing collaborative care vs usual care).

Yoshimatsu et al. 2023 consider as much as a 14-point change on the CDRS-R as a clinically relevant change (defined as a CGI-I score of 3, i.e., minimally improved). The authors emphasize that this is not, however, a difference one would typically expect between groups. The discussion concludes: “However, it seems to be difficult to expect a 14-point treatment difference of the CDRS-R for antidepressants compared with that with placebo, […] Based on our findings, the magnitude of the treatment difference for fluoxetine and fluoxetine with cognitive-behavioral therapy compared with that for placebo was half or more of the CDRS-R improvement, corresponding to minimal improvement in the CGI [our emphasis].” Given the latter, the smallest clinically relevant drop in CDRS-R that we have chosen (6 points) is close to what Yoshimatsu et al claim (7 points).

To sum this long answer (!), there are many different perspectives to consider, and there

does not seem to be one true answer. We will interpret the results of our study in relation to the methodological choices we made.

Thank you for this thorough answer! I completely agree that there does not seem to be one true answer when it comes to thresholds for clinically relevant change, and I think you show this and discuss it very well in this response. Why not include some of this discussion in the article? In my opinion, since it is such a difficult definition, a short discussion in the article of your choice versus other possible choices would be very interesting!

Thank you for a good suggestion. We have added some of the information from our response into the subheading "Power analysis" in the Methods section of the manuscript, see page 16 (lines 25-35).
